# Heavy Metal-Induced Variability in Leaf Nutrient Uptake and Photosynthetic Traits of Avocado (*Persea americana*) in Mediterranean Soils: A Multivariate and Probabilistic Modeling of Soil-to-Plant Transfer Risks

**DOI:** 10.3390/plants15020205

**Published:** 2026-01-09

**Authors:** Hatim Sanad, Rachid Moussadek, Abdelmjid Zouahri, Majda Oueld Lhaj, Houria Dakak, Khadija Manhou, Latifa Mouhir

**Affiliations:** 1 Laboratory of Process Engineering and Environment, Faculty of Science and Technology Mohammedia, University Hassan II of Casablanca, Mohammedia 28806, Morocco; majdaoueldlhaj1999@gmail.com (M.O.L.); latifa.mouhir@fstm.ac.ma (L.M.); 2 Research Unit on Environment and Conservation of Natural Resources, Regional Center of Rabat, National Institute of Agricultural Research, Rabat 10101, Morocco; abdelmjid.zouahri@inra.ma (A.Z.); houria.dakak@inra.ma (H.D.); 3 International Center for Agricultural Research in the Dry Areas (ICARDA), Rabat 10100, Morocco; r.moussadek@cgiar.org; 4 Laboratory of Natural Resources and Sustainable Development, Department of Biology, Faculty of Sciences, Ibn Tofail University, Kenitra 14000, Morocco

**Keywords:** HMs contamination, avocado (*Persea americana*), leaf nutrient uptake, photosynthetic performance, multivariate statistical analysis (MSA), Monte Carlo Simulation (MCS)

## Abstract

Soil contamination by heavy metals (HMs) threatens crop productivity, food safety, and ecosystem health, especially in intensively cultivated Mediterranean regions. This study investigated the influence of soil HM contamination on nutrient uptake, photosynthetic traits, and metal bioaccumulation in avocado (*Persea americana* Mill.) orchards. Twenty orchard sites were sampled, collecting paired soil and mature leaf samples. Soil physicochemical properties and HM concentrations were determined, while leaves were analyzed for macro- and micronutrients, photosynthetic pigments, and metal contents. Bioaccumulation Factors (BAFs) were computed, and multivariate analyses (Principal Component Analysis (PCA), Hierarchical Cluster Analysis (HCA), Linear Discriminant Analysis (LDA), and Partial Least Squares Regression (PLSR)) were applied to assess soil–plant relationships, complemented by Monte Carlo simulations to quantify probabilistic contamination risks. Results revealed substantial inter-site variability, with leaf Cd and Pb concentrations reaching 0.92 and 3.54 mg/kg, and BAF values exceeding 1 in several orchards. PLSR models effectively predicted leaf Cd (R^2^ = 0.789) and Pb (R^2^ = 0.772) from soil parameters. Monte Carlo simulations indicated 15–25% exceedance of FAO/WHO safety limits for Cd and Pb. These findings demonstrate that soil metal accumulation substantially alters avocado nutrient balance and photosynthetic efficiency, highlighting the urgent need for site-specific soil monitoring and sustainable remediation strategies in contaminated orchards.

## 1. Introduction

The rapid expansion of modern agriculture, driven by the need to feed a global population projected to reach 9.7 billion by 2050, has significantly intensified land use and agrochemical inputs [1]. While these practices have improved crop yields in the short term, they have also contributed to severe degradation of soil quality worldwide. Among the most alarming forms of soil degradation is the accumulation of toxic HMs, which threatens both agricultural productivity and food safety [2,3]. A recent global assessment estimates that approximately 14–17% of the world’s croplands, representing over 240 million hectares are contaminated with hazardous HMs, including cadmium (Cd), lead (Pb), nickel (Ni), arsenic (As), and chromium (Cr) [4]. This contamination poses direct and long-term risks to people reliant on these lands for food production and livelihoods [5]. HMs are particularly concerning because, unlike organic pollutants, they do not degrade over time and tend to accumulate in soils, persisting for decades or even centuries [6]. Their toxicity at trace concentrations, combined with their mobility in the soil–plant system, makes them among the most dangerous environmental pollutants. Major sources of agricultural soil contamination include atmospheric deposition from industrial emissions, mining activities, the over-application of chemical inputs, specifically phosphate fertilizers (which often contain Cd), untreated wastewater irrigation, and the disposal of sewage sludge on agricultural fields [7,8]. The rapid industrialization, urbanization and farming practices occurring in many developing regions, particularly across Asia, Africa, and parts of the Mediterranean, have further exacerbated the problem, with contaminated areas expanding each year [9].

HM contamination in agricultural soils presents a complex ecological and agronomic challenge, governed by multifaceted interactions between soil chemistry, plant physiology, and farming practices. Key soil properties, such as pH, organic matter content, cation exchange capacity (CEC), clay content, and redox conditions, critically regulate the solubility, mobility, and bioavailability of metals to plants [10,11]. For example, acidic soils tend to enhance the solubility of cadmium (Cd) and lead (Pb), facilitating their uptake by plant roots, whereas high organic matter content can immobilize these metals through complexation, thereby reducing their bioavailability [12,13]. These interactions are highly site-specific, shaped by local soil conditions, climatic regimes, and agricultural inputs, rendering broad regional assessments insufficient [14]. Instead, precise, site-specific evaluations are essential for accurately assessing contamination risks and designing sustainable soil management strategies [15]. Beyond direct effects on plant uptake, HM contamination has far-reaching implications for soil health. It disrupts fundamental soil functions such as nutrient cycling, microbial diversity, and structural stability, ultimately reducing land productivity and degrading agroecosystem resilience [16]. Importantly, the translocation of toxic metals into edible plant parts constitutes a significant food safety concern. The soil-to-plant transfer pathway is the principal route through which Cd, Pb, nickel (Ni), and zinc (Zn) enter the human food chain [17]. This uptake is influenced by both soil conditions, such as low pH, low organic matter, and high salt content and physiological mechanisms, including passive processes (mass flow and diffusion) and active transporters shared with essential nutrients [18]. Once absorbed, HMs accumulate in plant tissues, with leaves often serving as major sites due to their high transpiration rates and metabolic activity [19]. Additionally, HMs impair photosynthetic processes by interfering with chlorophyll biosynthesis and damaging photosystems I and II, reducing the efficiency of electron transport chains [20,21,22]. This can manifest as reduced chlorophyll a content and lower SPAD index values, which are commonly used proxies for assessing leaf photosynthetic performance and nitrogen status [23]. The accumulation of metals such as Cd and Pb stimulates the overproduction of reactive oxygen species (ROS), leading to lipid peroxidation, protein denaturation, and DNA fragmentation [24]. This oxidative damage compromises nutrient uptake efficiency and further diminishes photosynthetic capacity, ultimately lowering biomass production, fruit yield, and nutritional quality critical parameters for high-value crops like avocado (*Persea americana* Mill.) [25].

Among commercially important fruit crops, avocado has garnered substantial global attention due to its high nutritional value, economic profitability, and resilience to semi-arid climates [26]. Avocado production has grown rapidly in recent decades, reaching over 8 million tonnes globally in 2022, with significant cultivation expansion in Mediterranean regions, including Morocco, Spain, and Israel [27]. Avocado is recognized as a nutrient-dense fruit, rich in monounsaturated fats, dietary fiber, potassium, and bioactive compounds such as phytosterols and tocopherols, making it a key component of healthy diets worldwide [28]. However, this rapid production expansion often occurs in regions facing increasing environmental stresses, including water scarcity, soil degradation, and agrochemical overuse, raising concerns over its long-term sustainability [29,30]. Despite its increasing importance, avocado is relatively understudied with respect to its interactions with soil HM contamination [31]. Most existing research on HM uptake has focused on annual crops or cereals, with limited attention given to perennial fruit trees such as avocado. However, emerging evidence suggests that avocado is susceptible to HM accumulation under certain conditions, particularly in leaves and stems where metals such as Cd, Pb, and Ni can accumulate to levels that may impair physiological functions [32]. Studies indicate that elevated HM concentrations can alter nutrient uptake dynamics in avocado, leading to deficiencies in essential macronutrients like nitrogen (N), calcium (Ca), and magnesium (Mg), and micronutrients such as iron (Fe) and zinc (Zn) [33]. These disruptions may reduce chlorophyll synthesis, damage photosynthetic systems, and ultimately impair tree productivity and fruit quality.

The vulnerability of avocado to soil contamination is particularly relevant in the Mnasra region of Morocco, where avocado cultivation has expanded rapidly over the last two decades. This coastal plain, characterized by Mediterranean climatic conditions with semi-arid tendencies, has become a key production zone for avocado exports due to its favorable soil temperatures and access to groundwater for irrigation [34]. However, agricultural intensification in the Mnasra region has been accompanied by growing environmental pressure [2,35,36,37,38]. Previous studies in this region have documented elevated levels of soil HMs, attributed to both agricultural inputs and waste management practices [8,39]. However, research assessing the direct impacts of this contamination on avocado trees remains lacking. Specifically, no studies to date have simultaneously assessed leaf nutrient status, HM accumulation, and photosynthetic performance in avocado orchards exposed to contaminated soils in this region. This gap is critical, given the region’s economic reliance on avocado exports and the growing importance of maintaining both fruit quality and food safety standards for international markets. Additionally, few studies globally have explored how avocado trees respond physiologically to multi-element contamination under field conditions, particularly with respect to integrating soil chemistry, leaf tissue analysis, and photosynthetic traits.

Given the complexity of HM dynamics in agroecosystems, there is an increasing scientific consensus on the need for integrative, multidisciplinary approaches to better assess the risks of soil contamination on crop production and food quality. Traditional studies focusing solely on soil metal concentrations or leaf metal accumulation often fail to capture the full spectrum of interactions governing nutrient uptake, bioaccumulation, and physiological stress in plants [40,41]. A more comprehensive assessment requires simultaneous analysis of soil physicochemical properties, metal bioavailability, plant nutrient status, and functional physiological indicators such as photosynthetic efficiency. One of the most widely used metrics for assessing soil-to-plant metal transfer is the Bioaccumulation Factor (BAF) [42]. BAF provides a practical, site-specific indicator of the propensity of plants to accumulate metals from the soil and has been extensively applied in risk assessments for various food crops, particularly under field conditions [43]. However, few studies have applied BAFs specifically to perennial fruit trees like avocado, especially in regions affected by combined industrial, agricultural, and urban pollution sources. Furthermore, leaf photosynthetic indicators, such as the SPAD index (a proxy for chlorophyll content) and chlorophyll a and b concentrations have emerged as sensitive biomarkers of plant stress under HM exposure. Reductions in these indicators reflect disruptions in chlorophyll biosynthesis and damage to the photosynthetic apparatus caused by oxidative stress and nutrient imbalances [44]. Despite their diagnostic potential, such parameters remain underutilized in HM risk assessments for orchard systems, particularly in integrated models that account for both soil and plant factors.

Recent developments in multivariate statistical tools have greatly advanced our understanding of soil–plant interactions under HM stress. Exploratory methods such as PCA and HCA help reveal key patterns and site groupings based on soil and plant traits [45]. LDA further refines site classification based on physiological and bioaccumulation responses. However, these methods are primarily descriptive and offer limited predictive capabilities. To address this, PLSR has emerged as a robust predictive model capable of handling multicollinearity and multiple response variables simultaneously [46]. It enables the prediction of plant nutrient and physiological responses from soil HM concentrations. In parallel, Monte Carlo Simulation (MCS) offers a probabilistic framework to quantify variability and uncertainty in exposure assessments, especially for bioaccumulation and physiological impacts.

Addressing these scientific and practical gaps, the present study aims to provide a comprehensive, field-based assessment of the impacts of soil HM contamination on nutrient accumulation and photosynthetic performance in the leaves of avocado trees cultivated in the Mnasra region of Morocco. Specifically, this research seeks to:

(1) Assess and quantify the nutritional status of avocado leaves (macronutrients, micronutrients, and HMs) as well as their photosynthetic performance using SPAD index and chlorophyll a and b concentrations across multiple orchard sites;
(2) Evaluate the bioaccumulation capacity of avocado leaves for HMs through the calculation of Bioaccumulation Factors (BAFs);
(3) Investigate intra-specific variability in plant response and identify the key soil and physiological parameters driving differences among trees and sites using multivariate statistical techniques, including PCA, HCA, and LDA;
(4) Apply MCS to incorporate variability and uncertainty into ecological risk estimates, enhancing the robustness of risk prediction associated with HM accumulation;
(5) Develop predictive models using PLSR to quantify relationships between soil HM concentrations and leaf nutrient and photosynthetic traits.

## 2. Materials and Methods

### 2.1. Description of the Study Area

This survey was conducted in the Mnasra plain, a major agricultural zone located along the Atlantic coast of Morocco. Geographically, this region extends from the southern city of Kenitra to the banks of the Sebou River, with its eastern border marked by Sidi Allal Tazi and its northern limit reaching the Merja Zerga lagoon near Moulay Bouselham. The area is characterized by a Mediterranean climate, with an average annual precipitation of approximately 551 mm and moderate temperature fluctuations ranging between 12 °C in winter and 23 °C during the summer season [47]. Soils in the region predominantly consist of sandy, sandy clay, and silty clay textures, with sandy soils accounting for roughly 15% of the total land surface [38]. The agricultural economy of Mnasra revolves around market gardening, cereal production, and fruit orchards, supported by both mineral fertilizers such as ammonium nitrate, NPK blends, urea and organic amendments derived from cattle and poultry manure [47]. However, the region also faces significant environmental pressures from the Ouled Berjal landfill, which processes approximately 510 tons of mixed waste daily, including agricultural, industrial, and artisanal residues.

### 2.2. Sampling Design and Laboratory Analyses

#### 2.2.1. Leaf and Soil Sampling

**Figure 1:**
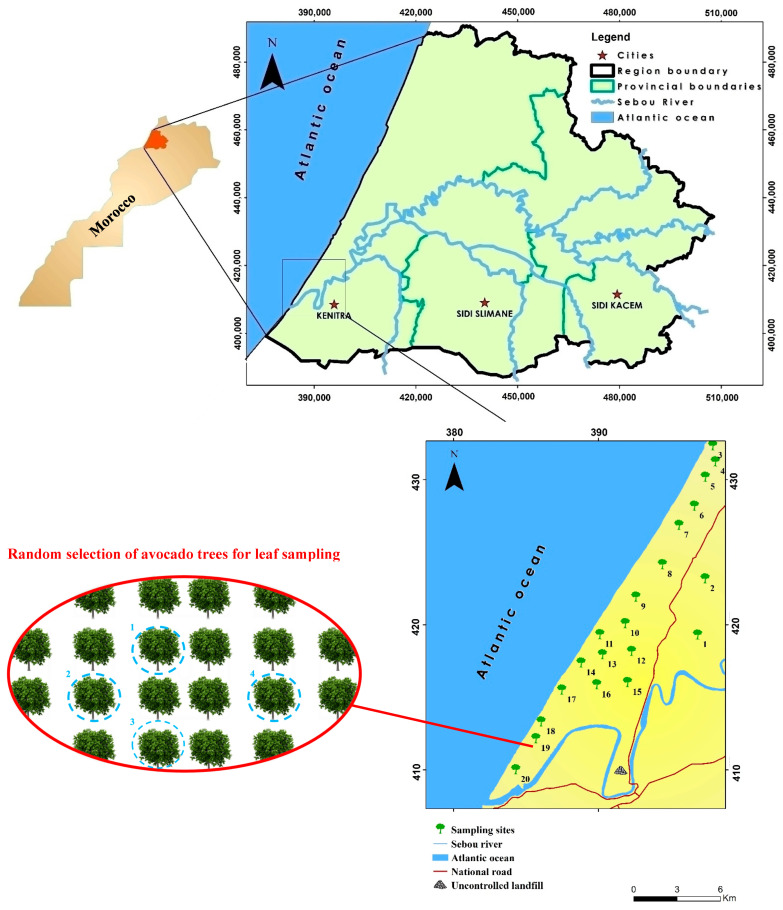
Map showing the location of soil samples and avocado orchards.

During 2024, systematic field sampling was conducted across the study area. Twenty distinct orchard sites were selected to represent the region’s spatial variability. In each site, four avocado trees planted in soils previously sampled for physicochemical and metal analyses were randomly selected (Figure 1). All sampled trees were in the harvest maturity stage to ensure comparability in physiological status. Leaf sampling followed the protocols recommended by the International Plant Nutrition Institute (IPNI) and the Food and Agriculture Organization (FAO) [48,49]. Fully expanded, healthy leaves were collected from the mid-canopy of each tree, avoiding older or damaged tissues. A minimum of 20 leaves per tree were harvested to ensure a representative sample, yielding an approximate fresh weight of 200 g per tree. The SPAD index was measured in the field using a SPAD-502 Plus chlorophyll meter, following the manufacturer’s guidelines. Measurements were taken on fully expanded, healthy leaves at the mid-canopy position, and the mean of five readings per tree was recorded to minimize variability. Samples were carefully placed in polyethylene bags, labeled, and stored in a cooled container during transport to the laboratory to preserve biochemical integrity.

The results of soil analyses, including physicochemical properties, nutrient status, and HM concentrations for each site, were obtained from previous studies conducted by Sanad et al. [38,47] and are presented in the Supplementary Materials of this study. These analyses provided essential baseline data for site characterization and statistical modeling.

It is important to note that, although these earlier works reported summary statistics of the same orchards, the soil samples used in the present study were collected concurrently with leaf sampling during the 2024 growing season. Both soil and leaf samples were taken from the same experimental plots and under identical environmental conditions to ensure accurate representation of soil-to-plant transfer and minimize temporal uncertainty in BAF estimation.

#### 2.2.2. Leaf Sample Preparation and Analytical Procedures

Upon arrival at the laboratory, the collected avocado leaf samples were carefully rinsed with deionized water to eliminate surface dust and contaminants. The samples were then oven-dried at 65 °C until a constant weight was achieved to ensure complete moisture removal, after which they were finely ground using a stainless-steel mill to obtain a homogenous powder suitable for analysis. Nitrogen (N) content was determined using the Kjeldahl method with an automated Kjeldahl analyzer (VELP Scientifica UDK 159). The analysis of Phosphorus (P), Potassium (K), Calcium (Ca), Magnesium (Mg), Boron (B), Iron (Fe), Zinc (Zn), Copper (Cu), Manganese (Mn), Nickel (Ni), Cadmium (Cd) and Lead (Pb) was conducted using ICP-MS after digestion using a solution of 2.25% Nitric acid and 0.5% Hydrochloric acid [50,51]. To evaluate the quantities of photosynthetic pigments, mature leaves were triturated in a mortar with 8 mL of methanol (90:10 *v*/*v*) for 1 min. The mixture was centrifuged at 4 °C at 6440× *g* for 5 min [52,53]. The supernatant was collected to quantify the content of chlorophyll a and chlorophyll b using Equations (1) and (2) established by Lichtenthaler and Buschmann [54].

(1) Chlorophyll a=16.82×A665.2−9.28×A652.4

(2) Chlorophyll b=36.92×A652.4−16.54×A665.2

The chlorophyll a/b ratio was calculated using the following Equation (3):

(3) $$\mathrm{Chlorophyll}\;a/b\;\mathrm{ratio}\;=\frac{Chlorophyll\;a}{Chlorophyll\;b}$$

### 2.3. Bioaccumulation Factor (BAF) Calculation

The Bioaccumulation Factor (BAF) is a widely used index to quantify the transfer of HMs from soil to plant tissues. It was calculated for each metal using the following Equation (4) [55,56]:

(4) $$Bioaccumulation\;Factor\;(BAF)=\frac{C_{Leaf}}{C_{Soil}}$$

where “C_Leaf_” represents the metal concentration in avocado leaf tissues (mg/kg) and “C_Soil_” corresponds to the metal concentration in the soil (mg/kg). This index provides a dimensionless value representing the metal accumulation capacity of the plant relative to soil metal availability.

### 2.4. Multivariate Statistical Analyses (MSA)

To evaluate the complex interactions between soil properties, HM concentrations, leaf nutrient status, and physiological traits of avocado trees, we employed both descriptive and multivariate statistical approaches. First, a descriptive analysis was conducted to summarize the distribution of each measured parameter across the 20 study sites. For each site, the mean values and standard error of the mean (SEM) were calculated for key variables, including soil nutrient contents, HMs, and leaf-level parameters. Next, to identify significant relationships between variables, Pearson’s correlation analysis was employed to quantify the direction and strength of linear associations between soil HMs, bioaccumulation, leaf nutrient profiles, and photosynthetic traits. To reduce dimensionality and explore latent structures in the dataset, PCA was applied. PCA was particularly useful in revealing dominant patterns of variability and the contribution of specific soil or leaf parameters to site differentiation. HCA was performed to classify sites based on their overall soil and plant trait profiles, allowing the identification of distinct clusters with shared characteristics. To further assess the robustness of these groupings, LDA was applied, which also served to identify the most discriminant variables between clusters [57]. To investigate the predictive relationships between soil contamination and plant response, Partial PLSR was conducted. PLSR enabled simultaneous modeling of multiple correlated response variables (leaf nutrients, HMs, and SPAD index) using soil HM concentrations as predictors, accounting for multicollinearity and high-dimensionality often present in environmental datasets [58]. To incorporate uncertainty and assess probabilistic risk, an MCS framework was developed. This simulation modeled variability in metal uptake and physiological stress under different soil scenarios by generating 10,000 iterations for each parameter, yielding probability distributions and confidence intervals.

### 2.5. Statistical Framework and Multivariate Testing Procedures

All datasets were screened for completeness and outliers before analysis. Descriptive statistics (mean ± SEM) were calculated for each parameter to evaluate within-site variability. To determine statistical differences among orchard sites, a one-way ANOVA was performed using the Welch test (to account for unequal variances), followed by Games–Howell post hoc comparisons to identify pairwise differences between sites. Significance thresholds were set at *p* < 0.05 (*), *p* < 0.01 (**), and *p* < 0.001 (***). To examine relationships among soil properties, BAFs and leaf biochemical and physiological traits, Pearson correlation analyses was employed depending on data distribution. Correlation coefficients (r) were interpreted following conventional strength categories (weak: 0.2–0.4, moderate: 0.4–0.6, strong: >0.6), with significance evaluated using two-tailed *p*-values. Correlation matrices were visualized through heatmaps showing r-values and significance levels (*, **, ***).

Multivariate analyses were conducted following standardized statistical protocols. PCA was applied using the covariance matrix with varimax rotation to identify the principal gradients driving variability and to reduce data dimensionality. HCA employed Ward’s linkage and Euclidean distance to classify orchard sites with similar soil–plant characteristics. LDA was used to validate the HCA clusters and to identify the most discriminant variables separating site groups based on Wilks’ lambda and F-to-enter criteria (*p* < 0.05). PLSR was implemented to predict leaf HM and nutrient concentrations from soil variables while addressing multicollinearity through latent variable extraction.

All statistical computations were carried out in XLSTAT 2024.1 (Addinsoft, Paris, France), whereas the MCS used for probabilistic risk modeling was implemented in Python 3.11 [2,37,59].

## 3. Results

### 3.1. Leaf Nutritional Status, Photosynthetic Pigments, and HM Accumulation in Relation to Soil Quality and Farming Practices in Avocado Orchard

#### 3.1.1. Macronutrient Dynamics in Avocado Leaves

All detailed values for each tree across all sites are provided in the Supplementary Materials. Specifically, Tables S4 and S5 present the macronutrient, micronutrient, and HM concentrations in avocado leaves for each sampled tree, while Tables S6 and S7 summarize the photosynthetic pigment profiles, SPAD index, chlorophyll a/b ratio, and leaf color status across orchard sites. Full descriptive statistics (mean and SEM) for each site are included in Table 1 and Table 2.

Leaf macronutrient concentrations showed moderate variability among the 20 avocado orchard sites (Table 1). N ranged from 2.23% (S9, S17) to 3.06% (S1), with an overall mean of 2.64%, indicating that most orchards maintained N levels above the optimal range of 1.8–2.4%, particularly sites S1, S5, S7, S14, S16, and S20, suggesting intensive fertilization inputs [60,61]. The lowest N contents were recorded in S17 (2.23%), S3 (2.24%), and S19 (2.30%), reflecting possible nutrient depletion in soils of these orchards. P concentrations varied between 0.25% (S2) and 0.40% (S20) (mean = 0.30%), slightly exceeding the sufficiency range of 0.08–0.25% for avocado leaves [61,62]. The highest P contents were observed in S20 (0.40%), S19 (0.38%), and S18 (0.32%), while lower values occurred in S2 (0.25%) and S17 (0.26%). K ranged from 1.58% (S5) to 2.71% (S1) (mean = 2.01%), indicating variable fertilizer application practices across orchards. Elevated K concentrations in S1, S7, S10, S11, and S20 suggest well-balanced soil fertility, whereas reduced values in S5, S4, and S9 may signal soil leaching or lower fertilizer availability [61,62]. Ca content spanned from 1.96% (S17) to 3.50% (S2 and S7) (mean = 2.77%), with the highest Ca levels associated with orchards having neutral-to-alkaline soils (S2, S5, S6, S7, S12), whereas lower Ca concentrations occurred in S14–S17, possibly reflecting acidic soil conditions or limited Ca mobility. Mg ranged from 0.46% (S3) to 0.86% (S7) (mean = 0.65%). The elevated Mg levels in S1, S7, and S20 correspond to favorable soil fertility and adequate nutrient supply, while the lowest values in S17 (0.47%) and S18 (0.50%) indicate nutrient stress likely linked to soil degradation and heavy-metal interference with Mg uptake [61,62]. Overall, these macronutrient patterns highlight strong site-specific variability in nutrient availability, with nutrient-rich orchards such as S1, S7, and S20 contrasting with nutrient-depleted sites S17, S18, and S3, emphasizing the influence of soil quality and management intensity on avocado leaf nutrient status.

#### 3.1.2. Micronutrient Uptake Patterns in Avocado Leaves and Soil Influences

Micronutrient concentrations in avocado leaves exhibited pronounced variability among orchard sites, reflecting differences in soil fertility and management conditions. Fe ranged from 91.20 mg/kg (S17) to 236.80 mg/kg (S20), with an overall mean of 157.3 mg/kg, generally within the adequate range for avocado (50–200 mg/kg). The highest Fe values were observed in S7, S1, and S20, which are characterized by higher soil organic matter and favorable pH, enhancing Fe bioavailability [61,62]. In contrast, the lowest Fe levels occurred in S17, S18, and S3, associated with sandy textures and degraded soil structure, limiting Fe uptake. Zn concentrations varied between 25.70 mg/kg (S17) and 80.00 mg/kg (S20) (mean = 42.7), with most sites within the optimal range (30–150 mg/kg). Higher Zn levels were detected in S10, S19 and S20, coinciding with balanced fertilization and moderate organic matter. Conversely, S3, S9, and S17 recorded suboptimal Zn values, likely due to low pH and limited Zn mobility. Cu showed moderate variation, from 7.38 mg/kg (S9) to 20.62 mg/kg (S20) (mean = 11.6 mg/kg). Most orchards fell within the recommended range (5–15 mg/kg), with higher Cu values in S19 and S20, possibly reflecting controlled Cu-based fungicide use [61,62]. Mn ranged from 10.00 mg/kg (S16 and S19) to 70.00 mg/kg (S2 and S7) (mean = 39.7 mg/kg). Elevated Mn concentrations coincided with healthy leaf coloration and favorable organic matter content, while lower Mn levels were associated with low organic matter and soil acidification. B concentrations ranged from 45.95 mg/kg (S8) to 55.35 mg/kg (S9) (mean = 51.4 mg/kg), with all sites falling within the adequate range (20–100 mg/kg). The highest B values reflected optimal irrigation and loamy soil textures, while lower levels likely resulted from leaching losses in sandy soils.

#### 3.1.3. HM Accumulation Patterns and Environmental Risks in Avocado Leaves

The concentrations of trace metals in avocado leaves exhibited notable variation across the 20 orchard sites, reflecting the combined influence of soil contamination levels, management intensity, and site-specific soil characteristics. Cd concentrations ranged from 0.11 mg/kg (S1, S7) to 0.31 mg/kg (S17), with a mean of 0.19 mg/kg, approaching the FAO/WHO upper permissible limit of 0.2 mg/kg for edible leafy tissues [63]. The highest Cd values occurred in S17, S18, and S3, consistent with soils exhibiting low organic matter and sandy textures that enhance Cd mobility. In contrast, sites such as S1, S7, and S20 showed minimal Cd accumulation, indicating lower soil contamination and better nutrient management. Pb concentrations ranged from 0.33 mg/kg (S7) to 1.38 mg/kg (S18), with an overall mean of 0.94 mg/kg. Although all measured values remained below the general FAO/WHO safety threshold of 2 mg/kg, several orchards (S3, S17, S18) showed relatively elevated Pb levels, suggesting cumulative effects from fertilizer residues, atmospheric deposition, or irrigation water contamination [64]. Conversely, orchards S1, S7 and S20 recorded the lowest Pb concentrations, corresponding with improved soil structure and lower anthropogenic inputs. Ni concentrations exhibited the highest variability among metals, ranging from 3.21 mg/kg (S17) to 5.00 mg/kg (S7, S19, S20), with a mean of 4.49 mg/kg. Elevated Ni levels were observed primarily in S3, S17, and S18, where sandy textures and low cation exchange capacities may enhance Ni solubility [65]. In contrast, sites with higher organic matter and neutral pH (S7, S20) exhibited reduced Ni uptake, reflecting limited bioavailability under these soil conditions. Overall, HM accumulation in avocado leaves revealed a clear gradient linked to soil degradation and agronomic practices. Orchards characterized by intensive fertilizer application, poor irrigation control, and limited organic matter management (S3, S17, S18) showed higher HM contents, whereas sites practicing balanced fertilization and organic amendments (S1, S7, S20) demonstrated markedly lower metal uptake. These findings emphasize the significance of soil quality and management practices in mitigating HM transfer to plant tissues and ensuring food safety in Mediterranean avocado production systems.

#### 3.1.4. Photosynthetic Response in Avocado Leaves and Interactions Between Nutritional Status, HMs, and Soil Properties

**Figure 2:**
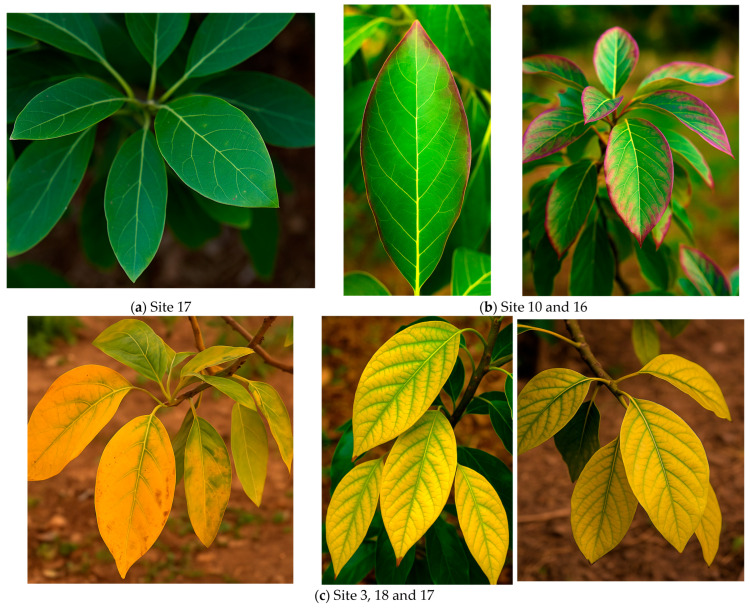
Leaf color and status (**a**) healthy green, (**b**) moderate (purple edges) and (**c**) yellowing at different survey sites.

Photosynthetic pigments and SPAD index values exhibited pronounced variability across orchard sites, revealing the close relationship between nutritional balance, HM stress, and leaf physiological performance [66]. The SPAD index ranged from 28.35 (S17) to 52.65 (S7), with a mean of 39.8, reflecting a gradient from chlorotic to healthy foliage [67]. The highest SPAD readings were recorded in S7, S20, and S1, where leaves maintained optimal N, Mg and Fe levels combined with low Cd and Pb contents. These trees exhibited “Healthy green” coloration and strong photosynthetic efficiency, supported by high chlorophyll a (≈ 65–70 µg/g FW) and balanced chlorophyll a/b ratios (~2.0–2.3) (Figure 2).

Conversely, S17, S18, and S8 showed the lowest SPAD values (25–30) and diminished chlorophyll a (20–27 µg/g FW) and chlorophyll b (12–18 µg/g FW) contents, consistent with “Yellowing/chlorotic” leaves. These orchards exhibited low Mg and Fe concentrations coupled with elevated Cd and Pb, conditions known to disrupt chlorophyll biosynthesis and electron transport. Intermediate SPAD values (~40–45) in orchards such as S4, S5, and S10 reflected moderate nutrient balance and partial pigment stress, often characterized by “purple edges” associated with phosphorus imbalance or transient HM exposure.

Chlorophyll a/b ratios ranged from 1.47 (S17) to 2.10 (S10), averaging 1.90 across sites. Ratios above 1.80 (e.g., S1, S7, S20) indicated efficient light-harvesting systems and minimal structural damage to chloroplasts, while ratios below 1.75 (e.g., S17, S18) suggested stress-induced chlorophyll degradation. Overall, photosynthetic traits displayed strong coherence with nutritional profiles and HM accumulation patterns: orchards with high nutrient availability and low HM stress maintained vigorous photosynthetic function, whereas sites affected by Cd and Pb contamination exhibited impaired pigment integrity and reduced photosynthetic efficiency.

### 3.2. Bioaccumulation Potential of HMs and Micronutrients in Avocado Leaves Across Orchard Sites and Trees

The Bioaccumulation Factor (BAF) is a key metric that quantifies the uptake efficiency of both essential micronutrients and non-essential toxic elements from soil to plant tissues. In this study, BAF results for individual trees across all orchard sites for seven elements, including Cd, Cu, Ni, Pb, Zn, Fe, and Mn, are presented in Table 3 and Table S8 in the Supplementary Data.

The results revealed substantial variability in BAF values both between and within sites, indicating that bioaccumulation is strongly modulated by soil properties, nutrient availability, and orchard management practices. Among all analyzed elements, Cd exhibited the highest variability, with BAF values ranging from 0.51 to 11.75 (mean: 3.52). Values exceeding 1 were widespread, confirming active Cd accumulation in many orchards. The highest value (11.75) was observed in Tree 2 of Site S5, characterized by sandy texture, low organic matter, and excessive phosphate fertilizer use, which collectively enhance Cd solubility and root uptake. Intra-site variability was also significant for example, in Site S3, BAFs ranged from 4.1 to 9.5, likely due to heterogeneity in root systems, soil microhabitats, and localized agrochemical inputs. In contrast, Fe and Mn, two essential micronutrients, exhibited consistently high BAF values across sites. Fe BAF ranged from 15.23 to 60.88 (mean: 36.26), and Mn from 6.67 to 51.02 (mean: 11.71), reflecting strong uptake potential. These high BAFs were especially notable in S4, S5, and S6, where slightly acidic soils and moderate organic matter content enhanced metal solubility. Within-site differences remained notable, as in Site S4, where Fe BAF varied between 30.2 and 55.7. Trees with higher Fe and Mn bioaccumulation generally showed better SPAD index, chlorophyll content, and leaf health status, reinforcing their physiological importance in photosynthesis and stress mitigation. Cu and Ni had consistently low BAFs across all orchard sites, with Cu ranging from 0.28 to 0.84 (mean: 0.52) and Ni from 0.06 to 0.12 (mean: 0.09). Their low bioavailability likely stems from strong sorption to soil colloids and limited solubility, particularly under conditions of neutral to alkaline pH and high organic matter. Minimal intra-site variability indicates that Cu and Ni uptake is predominantly restricted by uniform soil constraints across sites. Pb showed the lowest BAF values of all elements, with a narrow range from 0.006 to 0.059 (mean: 0.021). These values confirm the well-documented low mobility and bioavailability of Pb, which remains strongly bound to soil particles and is poorly translocated to above-ground plant parts. Notably, even in sites with elevated soil Pb such as S17 and S18, leaf Pb levels remained low, suggesting limited root-to-shoot transfer. Zn BAF values were moderate, ranging from 0.41 to 0.62 (mean: 0.51), and displayed greater inter-site variability than Cu and Ni. Trees from S7 and S20, which featured higher soil Zn levels, moderate organic matter, and loamy textures, exhibited relatively elevated Zn BAFs. Intra-site variability was limited, suggesting that Zn uptake was mainly driven by site-level conditions rather than tree-level variation. Overall, the BAF results highlighted distinct uptake patterns where high Cd, Fe, and Mn BAFs were found in degraded sites with acidic pH, sandy soils, and intensive chemical inputs (e.g., S3, S5 and S17). In contrast, optimal sites like S1, S7, and S20 showed low BAFs for toxic elements and moderate BAFs for essential micronutrients, reflecting balanced nutrient management and improved soil health. From a physiological perspective, Fe and Mn bioaccumulation correlated positively with SPAD index and chlorophyll levels, underlining their role in photosynthetic performance and oxidative protection. Conversely, elevated Cd BAFs were associated with reduced pigment levels and stress symptoms, aligning with Cd’s known toxic interference with chlorophyll biosynthesis and enzymatic activities.

### 3.3. Interrelationships Among Soil Properties, Leaf Nutritional Status, HM Accumulation, and BAF in Avocado Orchards

#### 3.3.1. Correlation Matrix

**Figure 3:**
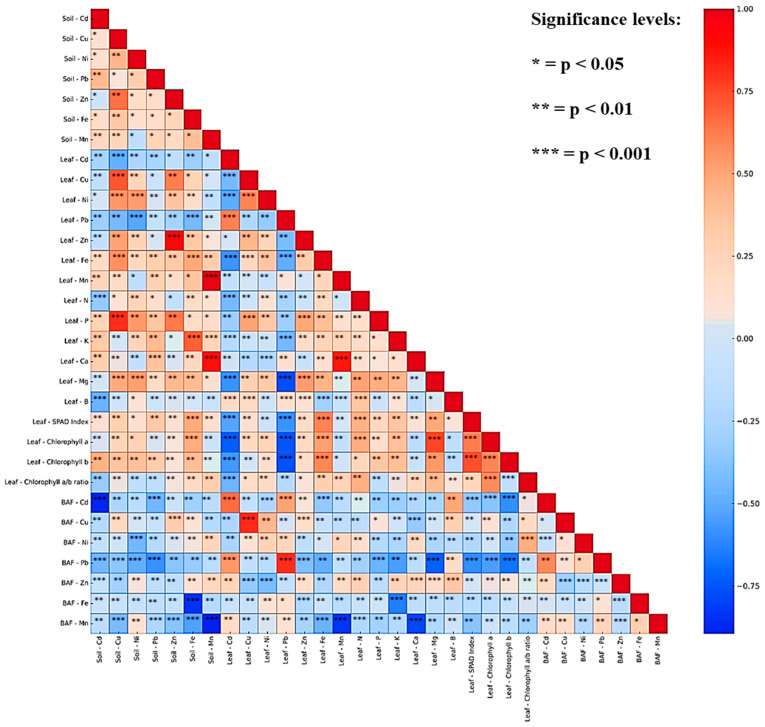
Correlation heatmap showing relationships among soil, leaf, BAFs and photosynthetic parameters in avocado orchards, cells display correlation coefficients (r) with significance levels.

To unravel the intricate relationships governing soil quality, leaf nutrient composition, metal accumulation, photosynthetic performance, and bioaccumulation behaviors in avocado trees, a comprehensive Spearman correlation analysis was conducted (Figure 3).

Strong positive correlations were observed between soil and leaf concentrations for certain elements, particularly Zn (r = 0.81), Ni (r = 0.60), and Cu (r = 0.58), demonstrating efficient root uptake and translocation of these metals. These results highlight the high mobility of Zn and Ni in avocado cultivation soils, which is consistent with their moderate solubility and known biological roles in plants. In contrast, elements such as Pb and Cd exhibited weak or non-significant soil-to-leaf correlations, suggesting physiological exclusion or restricted uptake, potentially due to root barrier mechanisms or soil immobilization under specific pH and organic matter conditions. A strong synergistic relationship was evident between leaf nutrients and photosynthetic traits.

P exhibited significant positive correlations with the SPAD index and both chlorophyll a and b levels, underscoring its critical role in ATP formation, energy transfer, and photosynthetic pigment synthesis [68]. N similarly showed strong associations with chlorophyll content, reflecting its fundamental role in chlorophyll molecule structure and Rubisco enzyme functionality [69]. Interestingly, micronutrients such as Fe and Zn in leaves were also positively linked to chlorophyll traits, suggesting their auxiliary roles in electron transport chains and antioxidant defense systems. Distinct behaviors emerged for BAF values across the orchards. For non-essential or potentially toxic metals such as Cd and Pb, BAF values tended to show weak or even negative correlations with their respective soil concentrations, suggesting potential saturation effects or physiological defense mechanisms that restrict metal accumulation at elevated soil concentrations. This pattern was especially evident for Cd, which showed high BAF variability but consistently weak links to soil levels, likely influenced by competition with essential nutrients such as Ca and Zn. Conversely, BAF values for essential micronutrients such as Fe and Mn were moderately correlated with both soil and leaf concentrations, indicating more consistent and regulated uptake processes.

#### 3.3.2. PCA of Soil, Leaf, BAF and Photosynthetic Parameters

**Figure 4:**
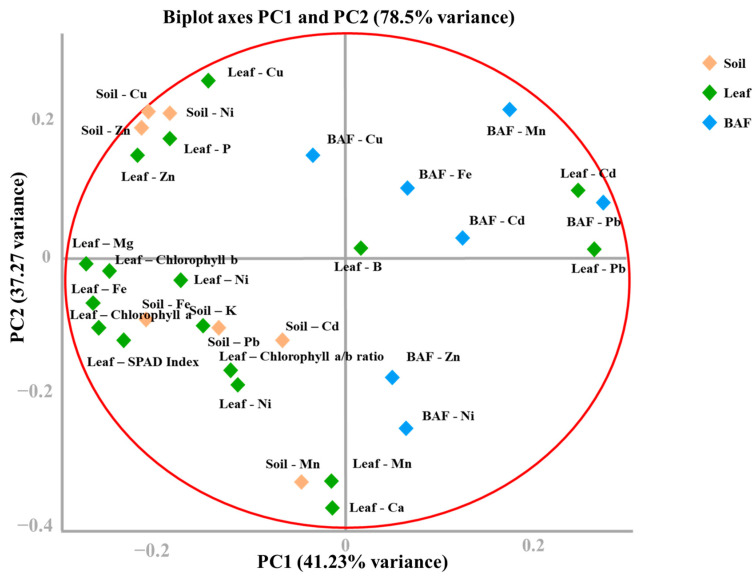
PCA results of soil, leaf, BAF and photosynthetic parameters.

To explore the multivariate interactions among soil properties, leaf nutrient composition, HM accumulation, BAF and photosynthetic performance, a PCA was conducted (Figure 4).

The first two principal components (PC1 and PC2) together explained 78.5% of the total variance, with PC1 accounting for 41.23% and PC2 for 37.27%. This high cumulative variance indicates that the two axes sufficiently captured the dominant environmental and physiological gradients across all orchard sites.

PC1 represented a “soil fertility–photosynthetic efficiency” gradient, characterized by strong positive loadings of soil and leaf Cu, Zn, and Ni, along with leaf macronutrients N and P and photosynthetic variables (SPAD index, chlorophyll a, and chlorophyll b). These loadings reflect the critical interplay between soil micronutrient availability and leaf physiological activity. Orchards with fertile soils and well-balanced nutrient management (e.g., S7, S1, and S20) clustered positively along PC1, reflecting high nutrient uptake, strong photosynthetic performance, and low metal stress.

PC2 represented a “bioaccumulation–stress response” gradient, dominated by BAF-Cd, BAF-Pb, and corresponding leaf Cd and Pb concentrations, with inverse loadings for beneficial micronutrients Fe and Mn. This component delineates the contrast between orchards experiencing elevated HM stress (e.g., S3, S5, and S17) and those maintaining micronutrient homeostasis. Negative loadings of Fe and Mn suggest competitive inhibition between essential and toxic metals under contaminated soil conditions, resulting in diminished chlorophyll synthesis and photosynthetic efficiency.

The PCA score plot distinctly separated orchard sites into two main clusters. Cluster I included high-performing orchards (S7, S1, S20) located along positive PC1 values, characterized by optimal nutrient uptake and strong photosynthetic vigor. Cluster II, comprising degraded and HM-stressed orchards (S3, S5, S17), was projected toward the upper quadrants of PC2, where Cd and Pb accumulation coincided with reduced photosynthetic pigment levels and SPAD index.

Overall, the PCA underscores two dominant gradients driving variability in avocado orchards: (1) a nutrient-driven axis associated with soil fertility and photosynthetic efficiency, and (2) a metal-stress axis governed by HM bioaccumulation and physiological inhibition. The strong association of leaf N, P, and chlorophyll with PC1 confirms the importance of nutrient management in maintaining photosynthetic health, while the loading of BAF-Cd and BAF-Pb on PC2 highlights the necessity of mitigating HM stress through soil remediation, organic amendments, and controlled fertilization practices.

#### 3.3.3. HCA of Orchard Sites Based on Integrated Soil, Leaf, Bioaccumulation, and Photosynthetic Characteristics

**Figure 5:**
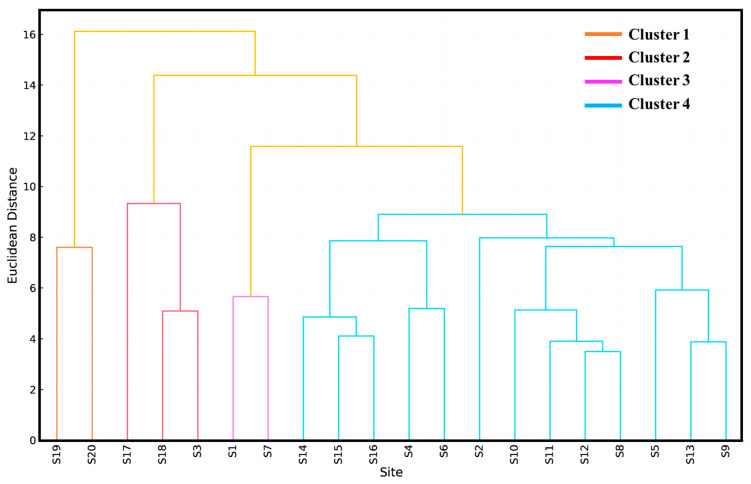
Dendrogram of orchard sites based on soil, leaf, BAF, and photosynthetic profiles.

To classify orchard sites based on integrated environmental and physiological parameters, HCA was performed using standardized Euclidean distance and Ward’s linkage method to generate robust site groupings. The resulting dendrogram distinguished four primary clusters, revealing consistent site-level patterns in soil–plant–physiology interactions (Figure 5).

The first group (Cluster 1) comprised Sites S19 and S20, characterized by balanced macronutrient profiles, moderate soil metal concentrations, low BAF values for toxic elements (Cd, Pb), and high SPAD and chlorophyll levels. These traits reflect efficient nutrient assimilation and optimal photosynthetic performance under relatively low metal stress, suggesting well-managed agronomic conditions. In contrast, Cluster 2 included Sites S17, S18, and S3, all of which experienced elevated Cd and Pb levels in soil and leaves, high BAFs, reduced macronutrient content, and significantly impaired photosynthetic performance. These sites clearly indicate HM-driven physiological stress and poor nutrient management, consistent with prior PCA results. Cluster 3, which grouped S1 and S7, represented the most productive orchards, exhibiting superior nutrient availability (notably N, P, Fe, Zn), minimal bioaccumulation risks, and the highest chlorophyll indices. Cluster 4 encompassed the majority of the remaining sites (S2, S4–S6 and S8–S16), forming a heterogeneous group marked by intermediate photosynthetic and nutritional profiles. These sites displayed localized HM accumulation, moderate BAF values, and variable nutrient status.

#### 3.3.4. Discriminant Analysis (LDA) of Orchard Sites Based on Integrated Soil, Leaf, BAF and Photosynthetic Parameters

**Figure 6:**
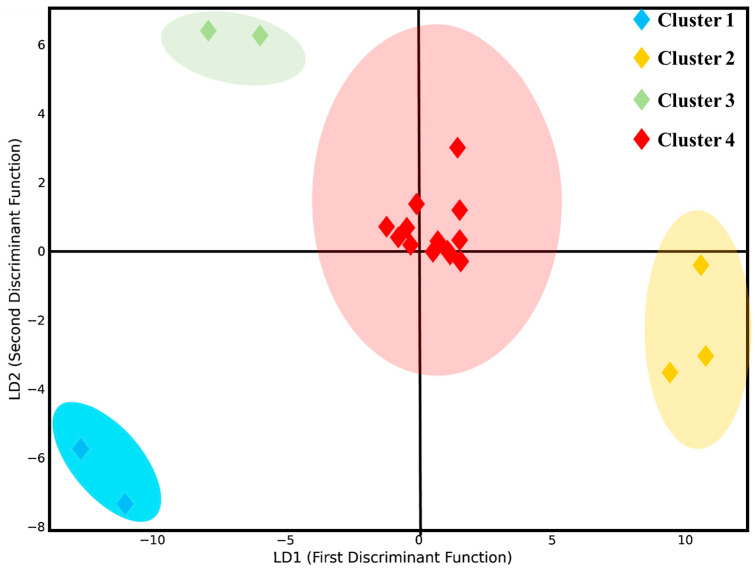
LDA results of orchard sites.

To statistically validate the orchard clusters identified through HCA and clarify the most influential variables responsible for site differentiation, an LDA was performed (Figure 6).

**Figure 7:**
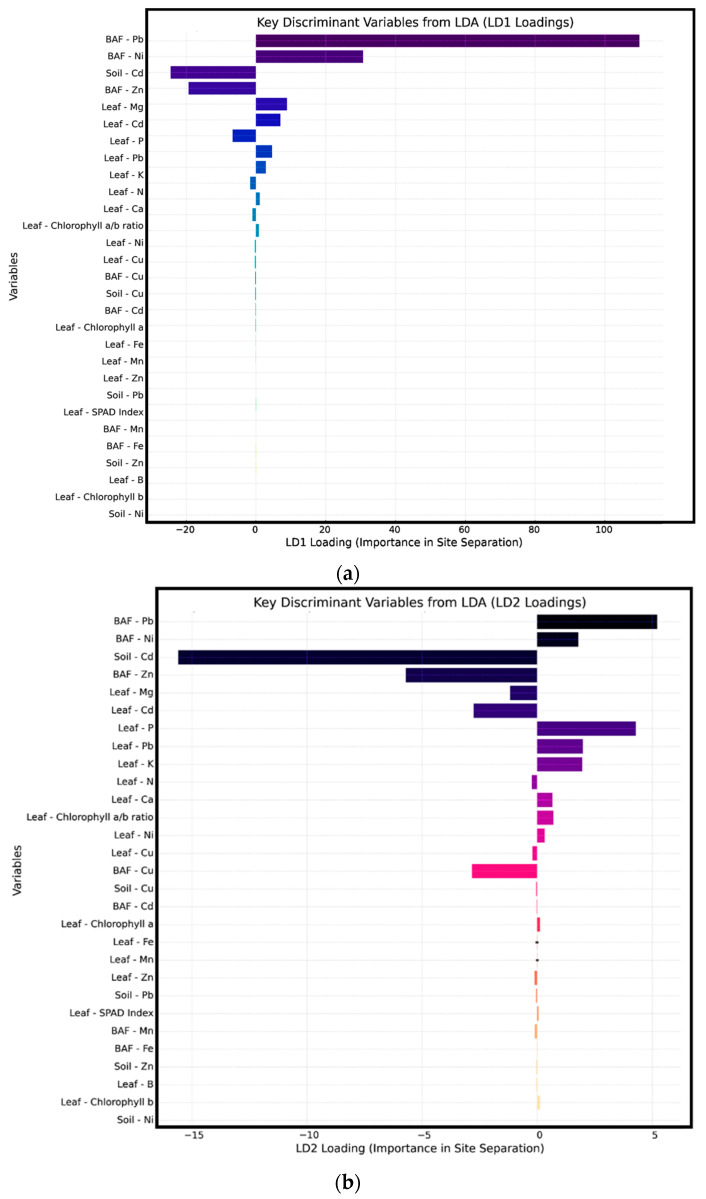
The key discriminant variables of (**a**) LD1 and (**b**) LD2.

The first two discriminant functions (LD1 and LD2) captured the majority of the inter-site variance, with LD1 effectively separating Cluster 1 (S19, S20) and Cluster 2 (S17, S18, S3) based on contrasting bioaccumulation and soil contamination profiles. Cluster 3 (S1, S7) differentiated primarily along LD2 due to elevated nutrient availability and photosynthetic performance, while Cluster 4, comprising the remaining sites, occupied a central position with intermediate traits. Key contributors to LD1 included the bioaccumulation factor for Pb (BAF–Pb), which had the highest discriminant loading, followed by BAF–Ni, BAF–Zn, and soil Cd, emphasizing the dominant role of HM stress in site segregation Figure 7. Leaf Cd and Mg also influenced differentiation, suggesting that tissue-level metal and nutrient balances are integral to site classification. LD2 added further nuance by highlighting the roles of soil Cd and leaf phosphorus, indicating stress-to-nutrient gradients. Overall, the LDA confirmed that metal bioaccumulation (especially Pb, Ni and Cd), soil contamination, and leaf nutrient status (notably Mg and P) are the most powerful discriminants of avocado orchard performance.

### 3.4. MCS of HM Bioaccumulation, Leaf Uptake, and Photosynthetic Traits Under Soil Variability

**Figure 8:**
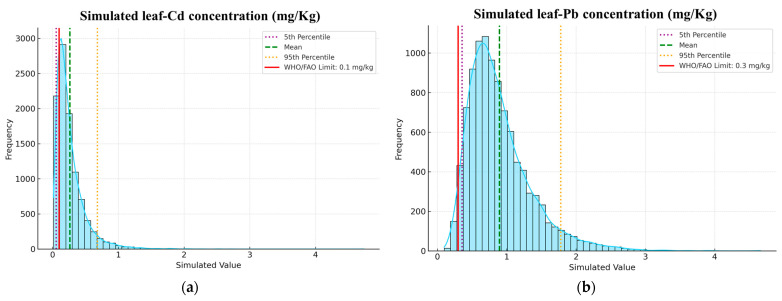
Probability distributions of (**a**) simulated leaf-Cd and (**b**) simulated leaf-Pb concentrations derived from MCS.

The simulation results revealed substantial variability across all modeled parameters (Figure 8 and Figure S1 in Supplementary Materials).

To assess the probabilistic risks of HM accumulation and associated physiological stress in avocado orchards, an MCS was conducted using log-normal distributions and 10,000 iterations per variable. This simulation incorporated key parameters identified in earlier multivariate analyses, including BAF–Cd, BAF–Pb, leaf concentrations of Cd and Pb, as well as SPAD index and chlorophyll a, in order to capture the full cascade of metal transfer from soil to leaf and its physiological consequences. The simulation revealed high uncertainty in Cd and Pb bioaccumulation, with Cd BAFs reaching a 95th percentile of 8.99, and Pb BAFs showing moderate variability. Simulated leaf concentrations raised critical food safety concerns with Cd exceeded WHO/FAO limits (>0.1 mg/kg DW) in over 95% of simulations, while Pb concentrations were consistently above safe thresholds (>0.3 mg/kg DW), indicating a substantial risk of contamination. On the physiological side, the simulated SPAD index (mean: 35.5) and chlorophyll a (mean: 45.4 µg/g FW) reflected moderate photosynthetic stress, consistent with metal-induced reductions in pigment synthesis and function. Overall, the MCS confirms a high probability of HM stress and reduced photosynthetic performance under current soil conditions.

### 3.5. Predictive Modeling of Leaf Nutrient Status and Photosynthetic Traits Using PLSR

**Figure 9:**
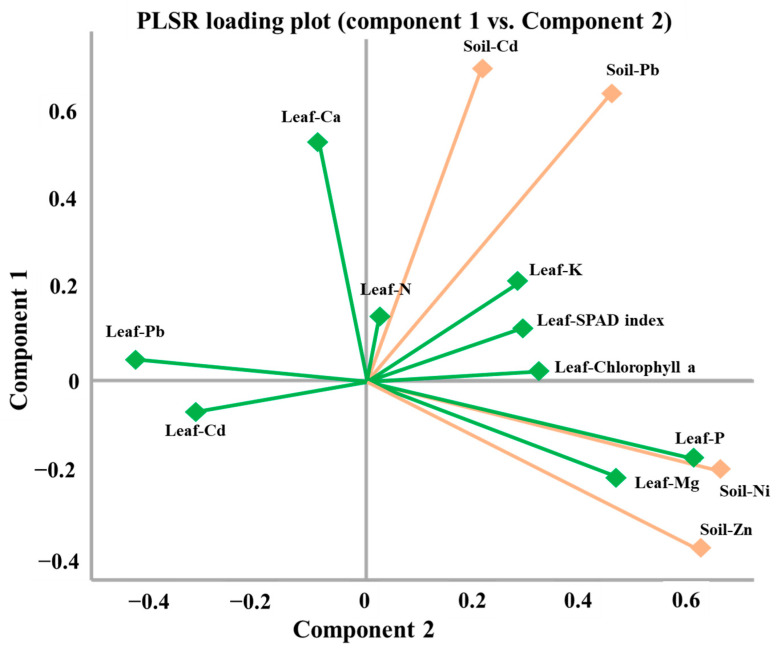
PLSR loading plot results.

To further elucidate the relationships between soil HHM contamination and leaf nutrient uptake and physiological performance, a PLSR analysis was performed. This method was selected for its robustness in modeling multicollinear data and its ability to simultaneously predict multiple response variables. The model utilized soil HM concentrations (Cd, Pb, Zn, and Ni) as predictor variables, while the response variables included leaf macronutrients (N, P, K, Ca, Mg), leaf HM concentrations (Cd, Pb), and photosynthetic indicators (SPAD index and chlorophyll a). The PLSR model, calibrated with two latent components, revealed varying degrees of predictive power across the different leaf parameters (Figure 9).

**Figure 10:**
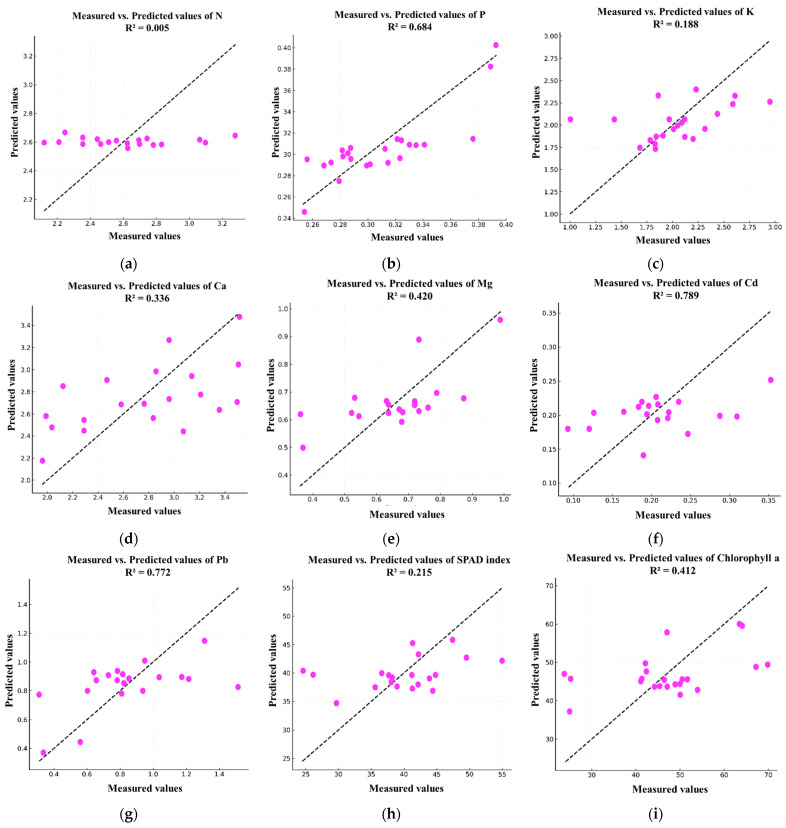
Detailed measured vs. predicted plots and associated R^2^ values for each leaf parameter (**a**) N, (**b**) P, (**c**) K, (**d**) Ca, (**e**) Mg, (**f**) Cd, (**g**) Pb, (**h**) SPAD index and (**i**) chlorophyll a.

The strongest predictive performance was observed for leaf HM concentrations, with R^2^ values of 0.789 and 0.772 for Cd and Pb, respectively (Figure 10).

These results clearly indicate that soil HM levels are highly effective predictors of metal accumulation in avocado leaves, consistent with the bioaccumulation patterns identified in prior analyses. The model also showed a moderate predictive ability for P (R^2^ = 0.684), Mg (R^2^ = 0.420), Ca (R^2^ = 0.336), and chlorophyll a (R^2^ = 0.412), suggesting that these plant traits are partially influenced by soil HM status.

However, the predictive capacity of the model was limited for N (R^2^ = 0.005), K (R^2^ = 0.188), and the SPAD index (R^2^ = 0.215). This indicates that these parameters are likely governed by other factors beyond soil HM concentrations, such as fertilizer management, irrigation practices, or microclimatic conditions.

Overall, this analysis highlights the utility of PLSR for predicting specific aspects of plant nutrient status and physiological performance from soil HM data. It demonstrates that while soil contamination strongly predicts metal accumulation in leaves, its influence on certain macronutrients and photosynthetic indicators is more limited. These findings emphasize the complexity of soil–plant interactions in contaminated agricultural systems and reinforce the need for integrative monitoring approaches.

## 4. Discussion

### 4.1. Integrated Discussion of Soil Quality, Bioaccumulation, Plant Uptake, and Photosynthetic Performance

This study provides an integrated understanding of how variations in soil metal contamination modulate nutrient uptake, bioaccumulation dynamics, and photosynthetic responses in avocado orchards across Mediterranean agroecosystems. The findings demonstrate a cascade effect, starting from spatial heterogeneity in soil metal concentrations (Cd, Pb, Zn, Ni) to differential bioaccumulation patterns (BAFs), ultimately impacting the leaf nutrient balance and photosynthetic functioning.

The observed variability in soil Cd and Pb levels was strongly associated with intensive agricultural inputs, particularly phosphate fertilizers and livestock manures, which are well-documented secondary sources of these metals [36,37,70]. Sites exhibiting degraded soil quality characterized by low organic matter content, coarse texture, and weak buffering capacity showed enhanced metal mobility and availability, facilitating plant uptake. Conversely, soils rich in organic carbon and with near-neutral pH exhibited lower HM solubility due to metal complexation and precipitation mechanisms [71]. These findings align with previous studies in Mediterranean fruit systems, where organic matter acts as a regulatory factor reducing bioavailable fractions of Cd and Pb [5,42,47].

BAFs for Cd and Pb emerged as dominant discriminant variables in both PCA and LDA analyses, underscoring their ecotoxicological significance and predictive value for soil-to-plant transfer. BAF values exceeding unity in multiple sites suggest active accumulation capacity in avocado leaves, reflecting either high soil-to-root transfer efficiency or translocation from roots to aerial tissues. This pattern agrees with previous evidence indicating that Cd and Pb can bypass exclusion mechanisms in perennial fruit trees through xylem transport, particularly under low Ca and Zn competition [32]. Furthermore, intra-site variability among trees indicates that root morphology, mycorrhizal interactions, and rhizosphere chemistry may modulate individual bioaccumulation responses, an aspect rarely captured in traditional site-level studies.

The physiological implications of metal stress were reflected in the SPAD index and chlorophyll content, which showed a consistent decline in metal-stressed orchards. Reduced SPAD and chlorophyll a levels in sites with high Cd and Pb accumulation suggest disruption of chlorophyll biosynthesis and photochemical efficiency, likely driven by oxidative stress, Mg substitution in chlorophyll molecules, and enzyme inhibition within the Calvin cycle [19,32]. In contrast, orchards with optimal macronutrient (N, P, Mg) and micronutrient (Fe, Mn) levels maintained high SPAD values and chlorophyll ratios, confirming the synergistic role of nutrient sufficiency in mitigating HM-induced stress. The positive correlation between SPAD, N, and Fe further supports the coupling between nitrogen metabolism and photosynthetic pigment formation under adequate soil fertility [72].

MCS strengthened the interpretation by quantifying probabilistic risks under field variability. The simulation indicated a 15–25% exceedance probability for Cd and Pb concentrations beyond FAO/WHO thresholds, confirming that current soil conditions pose measurable risks to both plant health and food safety. Moreover, modeled SPAD and chlorophyll distributions revealed moderate stress likelihood, reinforcing that even sub-toxic HM exposure can cause latent physiological impairments through cumulative stress. Such probabilistic modeling provides a more realistic assessment of contamination dynamics than deterministic averages, accounting for inherent environmental uncertainty.

Agronomic practices were identified as primary determinants of soil–plant interactions. Orchards managed with excessive agrochemical inputs, poor organic matter replenishment, and limited pH correction exhibited higher bioaccumulation and physiological stress. Conversely, balanced nutrient management, integration of compost or biochar, and regulated irrigation regimes were associated with enhanced soil buffering capacity, reduced metal uptake, and superior leaf pigment integrity. These findings align with sustainable orchard management frameworks emphasizing soil health as a cornerstone of plant resilience and yield quality [14,38].

Overall, the integrated interpretation of chemical, biological, and statistical evidence highlights a bidirectional linkage between soil contamination and avocado physiology. Soil degradation promotes HM mobility and plant stress, while improved nutrient and organic matter management reduces bioaccumulation risks and sustains photosynthetic performance. This reinforces the necessity for site-specific monitoring and adaptive soil remediation, combining nutrient balancing, organic amendments, and probabilistic risk modeling to ensure orchard sustainability and food safety in Mediterranean systems.

### 4.2. Recommendations and Mitigation Strategies for Reducing HM Risks and Enhancing Orchard Sustainability

Based on the comprehensive results of this study, several targeted recommendations can be proposed to mitigate HM risks in avocado orchards and improve overall soil and plant health. A first priority for high-risk sites involves reducing soil HM concentrations and minimizing metal bioavailability. The application of soil amendments such as biochar, compost, and organic manure can effectively immobilize HMs, particularly Cd and Pb, thereby reducing their uptake by plants. Biochar, known for its strong adsorption properties, not only binds metals but also enhances soil structure and water retention [73]. Additionally, the use of clay minerals and zeolite can further reduce metal mobility through ion exchange processes. In acidic soils, applying lime to increase pH can help immobilize Cd and Pb by transforming them into less soluble forms.

Optimizing nutrient management practices is another essential strategy for mitigating HM stress in avocado trees. Excessive application of phosphate fertilizers, often linked to Cd contamination, should be avoided. Instead, balanced fertilization plans tailored to site-specific nutrient needs are recommended to ensure adequate supply of N, P, Mg, and Fe without exacerbating metal uptake [74]. Moreover, foliar applications of key micronutrients such as Fe, Mn, and Zn can improve plant resistance to metal toxicity and maintain photosynthetic efficiency. The use of organic fertilizers with verified low metal content can also enhance nutrient availability while minimizing additional contamination risks [14].

Water quality management represents another critical aspect of reducing contamination risks. Regular monitoring of irrigation water for HMs is essential, especially in regions where water sources are vulnerable to industrial pollution or agricultural runoff [2,8]. In cases where contamination is detected, water treatment measures such as filtration systems or constructed wetlands can help reduce metal concentrations before irrigation [75]. In parallel, phytoremediation strategies may offer gradual, long-term solutions for reducing soil metal loads. Intercropping avocado trees with hyperaccumulator plants such as mustard, sunflower, or vetiver grass can assist in extracting metals from the soil, followed by safe removal of the plant biomass. Crop rotation and the use of cover crops can also improve soil organic matter content, enhance nutrient cycling, and mitigate HM accumulation in the soil over time [76].

Continuous monitoring and risk-based site management should be integral components of orchard management plans. Regular testing of leaf tissue for metal and nutrient content can serve as an early warning system for metal accumulation or nutritional imbalances [76]. Similarly, monitoring photosynthetic indicators such as the SPAD index and chlorophyll a levels can help detect physiological stress before yield losses occur [77]. Integrating these monitoring tools with risk assessment models, such as the MCS applied in this study, can support data-driven decision-making and enable precision management tailored to each orchard’s specific conditions [37].

Finally, broader actions at the regional and policy levels are necessary to support long-term orchard sustainability. Farmer education programs should be expanded to raise awareness about the risks of excessive agrochemical use and the importance of soil and water conservation practices. Subsidies or incentives for adopting soil amendments, organic fertilizers, and remediation technologies could encourage widespread adoption of best practices. In addition, governments should establish and enforce stricter regulatory limits for HMs in agricultural soils, irrigation water, and commercial fertilizers to prevent future contamination.

### 4.3. Study Limitations and Future Perspectives

While this study provides valuable insights into the soil–plant interactions governing metal dynamics in avocado orchards, certain limitations should be acknowledged. The research focused primarily on the soil–leaf interface to elucidate bioaccumulation patterns, nutrient-metal relationships, and physiological responses. Although this approach effectively characterizes the early indicators of contamination stress and plant health, it does not directly quantify the transfer of heavy metals to the edible fruit tissues, which are most relevant for assessing human exposure and food safety risks.

Future studies should therefore aim to integrate fruit metal analysis to establish a comprehensive soil–leaf–fruit transfer model, including the determination of translocation factors and bioavailability coefficients under diverse environmental and agronomic conditions. Additionally, longitudinal studies spanning multiple growing seasons could capture the temporal variability of metal accumulation and its relationship with orchard management practices, irrigation water quality, and climatic stressors. Incorporating advanced machine learning and geospatial modeling approaches could further enhance predictive accuracy for contamination risk mapping at the regional scale. Such integrative efforts will provide a more complete foundation for developing sustainable soil management and food safety strategies in Mediterranean horticultural systems.

## 5. Conclusions

The findings revealed significant spatial heterogeneity in both soil and leaf parameters, highlighting the intra-specific variability of avocado trees in response to local soil conditions. Elevated concentrations of Cd, Pb and Ni in soil were inversely correlated with essential nutrients (Mg, Ca, Fe) and photosynthetic indicators (SPAD index, chlorophyll a), suggesting that metal contamination compromises nutrient uptake and physiological performance. BAF analysis confirmed that avocado leaves can accumulate metals particularly Cd and Pb with marked variability across sites and individual trees, indicating site-specific differences in soil quality and management. These findings underscore the dual risk of soil degradation and potential food safety hazards in contaminated orchards.

Multivariate statistical analyses further elucidated the structure of these complex interactions. PCA and HCA identified distinct site clusters driven by combined soil–leaf–metal profiles, while LDA successfully discriminated among tree groups based on physiological and contaminant signatures. PLSR demonstrated strong predictive ability for leaf Cd and Pb concentrations from soil data (R^2^ > 0.77), while macronutrients such as N and K were less well-predicted, indicating that they are more strongly influenced by agronomic practices than by metal toxicity. MCS confirmed the probabilistic risk of Cd and Pb accumulation exceeding acceptable thresholds in certain locations, reinforcing the need for localized risk-based management strategies.

Overall, this study demonstrates that soil metal contamination primarily alters the metal accumulation dynamics and nutrient balance in avocado leaves, with secondary but measurable impacts on photosynthetic performance. The observed physiological alterations manifested as reductions in SPAD index and chlorophyll content reflect stress-induced interference with nutrient uptake and pigment biosynthesis rather than a direct inhibition of photosynthetic machinery. These findings emphasize that while agricultural management practices (fertilization and irrigation) remain the dominant determinants of avocado vigor, soil contamination acts as an aggravating stressor that reduces nutrient efficiency, weakens physiological resilience, and may compromise long-term orchard sustainability.

In sum, this research highlights the intricate interplay between soil contamination, plant nutrition, and physiological stress in avocado systems. The integration of bioaccumulation indices, multivariate modeling, and probabilistic simulations provides a robust and innovative framework for diagnosing and predicting contamination impacts in orchard environments, offering valuable insights for sustainable soil management and food safety in Mediterranean agro-ecosystems.

## Figures and Tables

**Table 1:** Site-level means (±SEM) and significance of leaf macronutrients, micronutrients, HMs and photosynthetic traits in avocado leaves across 20 orchard sites (S1–S10).

Site		Macronutrient (%)					Micronutrient (mg/kg)					HM (mg/kg)			Photosynthetic Pigment Profiles			
		N	P	K	Ca	Mg	B	Fe	Zn	Cu	Mn	Ni	Cd	Pb	SPAD Index	Chlorophyll a (µg/g FW)	Chlorophyll b (µg/g FW)	Chlorophyll a/b Ratio
S1	Mean	3.06	0.28	2.71	2.66	0.78	49.05	220.37	38.37	9.57	32.47	4.62	0.11	0.48	49.50	67.10	36.12	1.86
	SEM	0.11	0.01	0.09	0.10	0.02	3.64	11.32	2.85	0.74	1.98	0.18	0.01	0.04	1.48	0.70	1.08	0.07
	Sig.	**	**	**	*	**	*	*ns*	*	**	*	**	**	**	*	**	*	**
S2	Mean	2.46	0.25	2.04	3.50	0.60	49.80	137.05	45.05	7.85	70.00	4.24	0.20	0.76	42.75	42.77	26.70	1.61
	SEM	0.08	0.01	0.11	0.00	0.03	1.09	8.96	1.55	0.96	0.00	0.20	0.22	0.01	1.07	0.34	1.74	0.09
	Sig.	**	**	**	**	**	*	*ns*	*	**	**	**	**	**	*	**	*	**
S3	Mean	2.24	0.27	2.04	3.04	0.46	54.22	97.40	36.12	8.20	51.12	4.76	0.30	1.35	25.85	24.25	13.85	1.80
	SEM	0.08	0.01	0.18	0.08	0.06	1.91	6.03	0.96	0.95	4.32	0.09	0.01	0.06	1.38	1.36	1.08	0.23
	Sig.	**	**	**	**	**	*	*ns*	*	*	*	**	**	**	*	*	*	**
S4	Mean	2.70	0.28	1.63	2.76	0.62	51.67	144.57	39.67	10.5	35.67	4.48	0.19	0.95	39.07	47.75	24.75	1.94
	SEM	0.13	0.01	0.08	0.04	0.04	0.88	9.08	2.80	0.82	2.41	0.25	0.02	0.05	1.95	2.09	0.97	0.11
	Sig.	**	**	**	**	**	**	*ns*	*	**	*	**	**	**	*	*	**	**
S5	Mean	2.99	0.33	1.58	3.30	0.69	54.62	150.15	45.50	9.97	67.42	4.85	0.20	0.93	41.55	46.92	23.02	2.03
	SEM	0.17	0.01	0.20	0.08	0.06	2.16	2.09	2.40	0.71	1.57	0.08	0.01	0.02	1.64	2.42	1.34	0.01
	Sig.	**	**	**	**	**	*	*	*	**	*	**	**	**	*	*	*	**
S6	Mean	2.60	0.31	1.64	3.30	0.64	47.88	146.25	42.00	11.48	67.70	4.56	0.16	1.10	40.42	44.02	22.8	1.94
	SEM	0.06	0.01	0.22	0.08	0.06	1.49	10.54	2.98	0.61	1.50	0.26	0.02	0.06	1.42	3.33	0.94	0.15
	Sig.	**	**	**	**	**	*	*ns*	*	**	*	**	**	**	*	*	*	*
S7	Mean	2.65	0.32	2.28	3.50	0.86	48.92	225.20	41.12	9.05	70.0	5.00	0.11	0.33	52.65	65.68	33.48	1.98
	SEM	0.12	0.01	0.14	0.00	0.05	0.59	13.36	0.51	0.64	0.00	0.00	0.02	0.01	1.27	1.71	2.07	0.13
	Sig.	**	**	**	**	**	**	*ns*	**	**	**	**	**	**	*	*	*	**
S8	Mean	2.42	0.30	1.86	3.18	0.71	45.95	143.20	48.78	9.20	61.45	4.68	0.15	0.74	39.58	50.55	25.00	2.08
	SEM	0.07	0.01	0.14	0.12	0.02	2.37	12.69	2.22	1.05	3.13	0.32	0.02	0.07	1.26	1.59	2.06	0.23
	Sig.	**	**	**	**	**	*	*ns*	*	*	*	**	**	**	*	*	*	**
S9	Mean	2.23	0.27	1.60	2.74	0.64	55.35	144.20	34.58	7.38	38.17	4.16	0.16	1.02	40.67	50.10	26.05	1.92
	SEM	0.16	0.01	0.21	0.05	0.05	3.38	8.58	3.25	0.84	3.43	0.12	0.02	0.10	1.92	2.44	0.43	0.10
	Sig.	**	**	**	**	**	*	*ns*	*	**	*	**	**	**	*	*	**	**
S10	Mean	2.68	0.29	2.04	2.59	0.65	48.88	152.80	58.10	10.02	38.8	4.43	0.16	0.99	42.02	49.85	24.42	2.10
	SEM	0.12	0.01	0.08	0.11	0.05	2.89	9.66	3.23	1.08	0.63	0.33	0.01	0.08	1.23	1.88	2.11	0.25
	Sig.	**	**	**	**	**	*	*ns*	*	*	**	**	**	**	*	*	*	**

Note: Sig. = significance levels were determined using Welch’s ANOVA with Games–Howell post hoc test *ns* = non-significant (*p* ≥ 0.05), * = significant (*p* < 0.05), ** = highly significant (*p* < 0.01).

**Table 2:** Site-level means (± SEM) and significance of leaf macronutrients, micronutrients, HMs and photosynthetic traits in avocado leaves across 20 orchard sites (S11–S20).

Site		Macronutrient (%)					Micronutrient (mg/kg)					HM (mg/kg)			Photosynthetic Pigment Profiles			
		N	P	K	Ca	Mg	B	Fe	Zn	Cu	Mn	Ni	Cd	Pb	SPAD Index	Chlorophyll a (µg/g FW)	Chlorophyll b (µg/g FW)	Chlorophyll a/b Ratio
S11	Mean	2.55	0.30	2.33	3.24	0.62	50.30	166.12	52.88	9.38	58.52	4.52	0.19	0.83	41.18	48.22	24.80	1.98
	SEM	0.10	0.01	0.17	0.09	0.03	3.44	9.82	3.14	0.80	2.50	0.20	0.02	0.05	1.71	1.37	1.91	0.16
	Sig.	**	**	**	**	**	*	*ns*	*	**	*	**	**	**	*	*	*	**
S12	Mean	2.50	0.30	2.02	3.30	0.67	52.90	152.48	47.78	10.55	67.12	4.35	0.19	0.73	38.33	46.50	24.38	1.91
	SEM	0.03	0.00	0.16	0.13	0.03	1.53	10.54	1.67	0.55	1.29	0.28	0.02	0.04	1.33	0.87	0.91	0.04
	Sig.	**	**	**	**	**	*	*ns*	*	**	*	**	**	**	*	**	**	**
S13	Mean	2.61	0.29	1.88	2.99	0.64	47.80	129.30	44.78	8.52	49.65	4.80	0.17	0.83	39.78	47.15	24.42	1.93
	SEM	0.12	0.01	0.10	0.04	0.03	3.97	2.33	2.24	1.03	3.00	0.09	0.03	0.03	1.76	0.77	0.38	0.02
	Sig.	**	**	**	**	**	*	*	*	*	*	**	**	**	*	*	*	*
S14	Mean	2.90	0.29	1.88	2.23	0.62	51.92	150.93	42.50	8.07	12.6	4.62	0.20	0.93	41.08	47.40	26.02	1.88
	SEM	0.10	0.01	0.18	0.10	0.06	2.96	11.79	3.13	1.02	1.18	0.04	0.01	0.13	1.48	3.31	2.02	0.28
	Sig.	**	**	**	**	**	*	*ns*	*	*	*	**	**	**	*	*	*	**
S15	Mean	2.52	0.29	1.98	2.22	0.68	49.78	148.32	41.02	8.20	11.18	4.70	0.20	0.69	37.45	47.65	25.05	1.92
	SEM	0.14	0.02	0.12	0.08	0.02	2.82	9.49	1.38	1.92	1.17	0.21	0.03	0.05	1.09	2.21	1.23	0.16
	Sig.	**	**	**	**	**	*	*ns*	*	*	*	**	**	**	*	*	*	**
S16	Mean	2.80	0.27	2.10	2.26	0.66	50.28	144.95	43.48	8.30	10.00	4.94	0.17	0.86	37.83	45.80	25.15	1.84
	SEM	0.10	0.00	0.07	0.14	0.02	1.79	11.63	1.52	0.73	0.00	0.04	0.02	0.09	1.47	1.42	1.09	0.12
	Sig.	**	**	**	**	**	*	*ns*	*	**	**	**	**	**	*	*	*	**
S17	Mean	2.23	0.26	1.82	1.96	0.47	49.58	91.20	25.70	8.12	11.48	3.21	0.31	1.37	28.35	23.38	16.18	1.47
	SEM	0.07	0.01	0.21	0.08	0.04	3.18	5.22	0.66	0.64	1.41	0.19	0.02	0.14	0.97	1.26	1.43	0.13
	Sig.	**	**	**	**	**	*	*ns*	**	**	*	**	**	**	**	*	*	**
S18	Mean	2.36	0.32	1.79	2.70	0.50	53.70	101.50	42.68	10.55	30.52	4.85	0.29	1.38	26.78	24.25	15.00	1.72
	SEM	0.06	0.01	0.15	0.10	0.04	1.79	3.79	1.12	1.03	3.18	0.15	0.01	0.09	1.04	0.87	1.93	0.28
	Sig.	**	**	**	**	**	*	*	*	*	*	**	**	**	*	**	*	**
S19	Mean	2.30	0.38	1.64	2.04	0.60	54.22	153.98	55.92	18.60	10.00	5.00	0.16	0.66	39.12	47.10	25.05	1.90
	SEM	0.10	0.01	0.20	0.14	0.04	1.92	8.48	2.00	0.86	0.00	0.00	0.01	0.08	1.26	2.09	1.76	0.06
	Sig.	**	**	**	**	**	*	*ns*	*	**	**	**	**	**	*	*	*	**
S20	Mean	2.79	0.40	2.38	2.26	0.81	51.3	236.80	80.00	20.62	22.00	5.00	0.13	0.51	50.38	66.18	35.38	1.89
	SEM	0.09	0.00	0.08	0.11	0.06	1.67	9.42	0.00	1.17	3.58	0.00	0.01	0.06	1.14	1.42	1.77	0.12
	Sig.	**	**	**	**	**	*	*ns*	**	*	*	**	**	**	*	*	*	**

Note: Sig. = significance levels were determined using Welch’s ANOVA with Games–Howell post hoc test *ns* = non-significant (*p* ≥ 0.05), * = significant (*p* < 0.05), ** = highly significant (*p* < 0.01).

**Table 3:** Mean and Standard Error of the BAFs of HMs in avocado leaves across 20 orchard sites.

Site	BAF-Cd		BAF-Cu		BAF-Ni		BAF-Pb		BAF-Zn		BAF-Fe		BAF-Mn	
	Mean	SEM	Mean	SEM	Mean	SEM	Mean	SEM	Mean	SEM	Mean	SEM	Mean	SEM
S1	1.31	0.06	0.53	0.04	0.09	0.01	0.01	0.01	0.51	0.03	17.74	0.91	9.89	0.60
S2	1.54	0.17	0.56	0.06	0.09	0.01	0.01	0.01	0.50	0.01	31.79	2.08	8.17	0.01
S3	4.32	0.17	0.63	0.07	0.09	0.01	0.02	0.01	0.50	0.01	26.81	1.66	9.88	0.83
S4	3.94	0.54	0.58	0.04	0.10	0.01	0.01	0.01	0.48	0.03	50.78	3.19	9.86	0.66
S5	6.71	0.54	0.49	0.03	0.09	0.01	0.01	0.01	0.47	0.02	52.88	0.73	10.02	0.23
S6	1.78	0.19	0.49	0.02	0.10	0.01	0.02	0.01	0.51	0.03	43.09	3.10	10.11	0.22
S7	0.87	0.15	0.43	0.03	0.08	0.01	0.01	0.01	0.50	0.01	32.45	1.92	7.88	0.01
S8	2.20	0.27	0.51	0.05	0.10	0.01	0.01	0.01	0.52	0.02	31.88	2.82	10.20	0.52
S9	5.20	0.77	0.48	0.05	0.09	0.01	0.02	0.01	0.51	0.04	41.85	2.49	10.16	0.91
S10	5.47	0.45	0.52	0.05	0.10	0.01	0.02	0.01	0.53	0.03	22.17	1.40	10.46	0.17
S11	2.67	0.26	0.46	0.03	0.10	0.01	0.02	0.01	0.49	0.02	24.24	1.43	9.46	0.40
S12	2.33	0.30	0.50	0.02	0.09	0.01	0.02	0.01	0.55	0.01	27.62	1.90	9.55	0.18
S13	8.42	1.41	0.44	0.05	0.09	0.01	0.02	0.01	0.53	0.02	22.68	0.40	9.16	0.55
S14	1.84	0.05	0.45	0.05	0.09	0.01	0.02	0.01	0.51	0.03	46.36	3.62	12.04	1.12
S15	4.02	0.51	0.49	0.11	0.09	0.01	0.02	0.01	0.50	0.01	51.07	3.26	11.39	1.19
S16	4.18	0.48	0.49	0.04	0.09	0.01	0.02	0.01	0.55	0.01	49.13	3.94	12.83	0.01
S17	5.13	0.41	0.59	0.04	0.09	0.01	0.04	0.01	0.47	0.01	51.38	2.93	37.29	4.58
S18	4.13	0.08	0.56	0.05	0.08	0.01	0.03	0.01	0.50	0.01	25.21	0.91	9.43	0.98
S19	1.75	0.12	0.53	0.02	0.06	0.01	0.01	0.01	0.47	0.01	53.11	2.92	14.46	0.01
S20	2.51	0.28	0.53	0.03	0.07	0.01	0.01	0.01	0.42	0.01	22.87	0.91	11.93	1.94

## Data Availability

The data is available on request from the corresponding author.

## Supplementary Materials

The following supporting information can be downloaded at: https://www.mdpi.com/article/10.3390/plants15020205/s1, Table S1: Soil physical properties of the sampling sites in the survey area; Table S2: Soil chemical properties of the sampling sites in the survey area; Table S3: HM content (mg/kg) in selected samples; Table S4: macronutrient, micronutrient, and HM concentrations in avocado leaves across orchard sites (S1 to S10); Table S5: Macronutrient, micronutrient, and HM concentrations in avocado leaves across orchard sites (S11 to S20); Table S6: Photosynthetic pigment profiles, spad index, chlorophyll a/b ratio, and leaf color status of avocado leaves across orchard sites (s1 to s10); Table S7: Photosynthetic pigment profiles, spad index, chlorophyll a/b ratio, and leaf color status of avocado leaves across orchard sites (s11 to s20); Table S8: BAFs of HMs in avocado leaves across of 20 orchard sites; Figure S1: Probability distributions of simulated BAFs and photosynthetic indicators derived from MCS.
