# Supplementary material for: Heavy Metal-Induced Variability in Leaf Nutrient Uptake and Photosynthetic Traits of Avocado (Persea americana) in Mediterranean Soils: A Multivariate and Probabilistic Modeling of Soil-to-Plant Transfer Risks
Source: Plants (Basel). 2026 Jan 9;15(2):205. doi: 10.3390/plants15020205 (PMC12844934; doi:10.3390/plants15020205)
Supplement: Supplementary file 1 [file plants-15-00205-s001.zip › plants-3952185-supplementary.pdf]

Supplementary material

The supplementary material accompanying this research article provides detailed soil data essential to the understanding and replication of our findings. It includes comprehensive information on the physical, chemical, and heavy metal properties of the soils from 20 selected sampling sites (S1 to S20). These sites correspond to a subset (S8 to S27) of the 30 soil samples previously analyzed and published in the related research article by (Sanad et al., 2025c, 2024c). The included data cover soil texture classification, moisture content, bulk and particle densities, porosity, pH, electrical conductivity, organic matter, cation exchange capacity (CEC), macro- and micronutrient content, and concentrations of key heavy metals (e.g., Cd, Pb, Ni, Zn, Cr, Cu, As). These values served as the foundational input for site characterization, bioaccumulation assessments, multivariate statistical analyses, and predictive modeling conducted throughout this study.

Table S1. Soil physical properties of the sampling sites in the survey area.

Samples	Sand (%)	Silt (%)	Clay (%)	Textural class	Moisture (%)	Bulk Density (g/m^3)	Particle density (g/m^3)	Total Porosity (%)	CaCO3 (%)
S1	21.00	57.71	21.29	Silty loam	11.40	1.39	2.30	39.57	1.27
S2	90.21	6.86	2.93	Sand	1.80	1.58	2.80	43.57	0.00
S3	87.86	7.57	4.57	Sand	2.50	1.55	2.60	40.38	0.00
S4	89.79	2.79	7.43	Sand	1.50	1.56	2.60	40.00	0.45
S5	88.29	5.14	6.57	Sand	3.30	1.55	2.60	40.38	0.30
S6	90.21	2.86	6.93	Sand	1.30	1.57	2.80	43.93	0.00
S7	90.07	7.93	2.00	Sand	3.20	1.57	2.80	43.93	0.00
S8	88.07	0.43	11.50	Loamy sand	3.70	1.49	2.50	40.40	0.00
S9	89.64	5.43	4.93	Sand	0.90	1.52	2.60	41.54	0.00
S10	91.36	5.43	3.21	Sand	3.60	1.59	2.80	43.21	0.54
S11	92.36	5.14	2.50	Sand	3.89	1.59	2.80	43.21	0.27
S12	83.36	11.93	4.71	Loamy sand	5.25	1.54	2.60	40.77	0.27
S13	81.00	9.14	9.86	Loamy sand	4.79	1.53	2.60	41.15	0.00
S14	79.86	7.79	12.36	Sand loam	4.71	1.49	2.50	40.40	0.00
S15	81.79	8.71	9.50	Loamy sand	3.96	1.53	2.60	41.15	0.00
S16	90.07	5.86	4.07	Sand	5.26	1.58	2.80	43.57	0.54
S17	88.14	7.14	4.71	Sand	2.69	1.55	2.60	40.38	0.56
S18	86.00	5.64	8.36	Loamy sand	3.20	1.52	2.60	41.54	0.00
S19	86.21	7.50	6.29	Loamy sand	2.28	1.55	2.60	40.38	0.00
S20	87.50	10.21	2.29	Sand	8.14	1.56	2.70	42.22	0.27
Mean	84.13	9.06	6.80	-	3.86	1.54	2.64	41.58	0.22
SEM	3.41	2.62	1.02	-	0.54	0.01	0.02	0.32	0.07

Table S2. Soil chemical properties of the sampling sites in the survey area.

Samples	pH	EC (dS/m)	OM (%)	CEC (cmol/kg)	Macronutrients and Macronutrients of soils (mg/kg)									
					**Av. N**	**Av. P**	**Av. K**	**Na**	**Ca**	**Mg**	**Fe**	**Zn**	**Cu**	**Mn**
S1	7.86	0.38	5.90	2.44	148.30	6.02	195.00	4.55	28.00	4.00	12.42	75.00	18	3.28
S2	7.04	0.28	1.90	2.22	123.40	17.23	105.00	4.37	5.50	3.50	4.31	89.00	14	8.56
S3	7.48	0.16	1.90	1.59	103.07	12.62	220.00	4.58	6.00	7.50	3.63	71.00	13	5.17
S4	7.45	1.21	3.43	1.34	89.04	10.86	120.00	4.12	19.50	4.00	2.85	83.00	18	3.62
S5	7.89	3.05	4.95	2.44	75.01	23.83	90.00	4.12	6.00	8.50	2.84	95.00	20	6.73
S6	7.08	0.91	3.14	2.32	75.01	18.17	130.00	4.43	9.00	4.50	3.39	82.00	23	6.69
S7	6.75	0.68	2.85	2.22	71.86	20.65	255.00	4.71	5.50	7.00	6.94	81.00	21	8.88
S8	7.45	1.04	2.09	2.32	85.89	29.62	355.00	4.47	13.50	*bdl*	4.49	93.00	18	6.02
S9	7.05	0.29	1.90	2.21	82.74	1.30	80.00	3.86	9.00	4.00	3.45	67.00	15	3.75
S10	7.56	0.79	3.62	1.83	68.71	52.15	325.00	5.01	10.00	4.50	6.89	109.00	19	3.71
S11	7.47	0.31	3.43	1.83	93.62	49.08	112.50	4.77	10.00	1.50	6.85	108.00	20	6.19
S12	7.18	0.21	2.47	1.81	79.59	37.28	72.50	5.26	8.00	1.00	5.52	86.00	21	7.03
S13	6.61	0.22	2.28	1.79	65.56	35.40	97.50	5.06	6.00	2.00	5.70	83.00	19	5.42
S14	7.55	0.29	4.57	1.68	110.80	13.10	100.00	6.54	5.50	1.50	3.26	82.00	18	1.05
S15	7.69	0.49	2.85	1.61	82.74	31.03	102.50	5.07	10.00	9.50	2.90	81.00	17	0.98
S16	7.71	0.37	4.00	1.61	79.59	40.00	125.00	5.55	13.50	*bdl*	2.95	79.00	17	0.78
S17	7.68	0.26	1.62	1.46	68.71	9.32	80.00	5.01	18.00	2.00	1.78	55.00	14	0.31
S18	8.08	0.30	1.86	1.71	71.86	4.25	65.00	5.47	7.50	*bdl*	4.03	85.00	19	3.23
S19	7.15	0.23	2.09	1.57	85.89	8.85	70.00	4.96	5.00	*bdl*	2.90	117.00	35	0.69
S20	7.33	1.09	3.33	2.40	106.22	79.05	275.00	4.99	15.50	3.00	10.35	190.00	38	1.84
Mean	7.44	0.62	3.01	1.92	88.38	24.99	148.75	4.84	10.55	3.40	4.87	90.55	19.85	4.19
SEM	0.08	0.14	0.26	0.08	4.67	4.37	20.01	0.13	1.33	0.64	0.60	6.14	1.39	0.60

Note: bdl (below detection limit), Mg detection limit = 0.2 mg/kg (based on the method used: AAS using novAA 800 D Analyzer).

Table S3. HM content (mg/kg) in selected samples.

Samples	As	Cd	Cr	Cu	Ni	Pb
S1	28,00	0,09	284,00	18,00	51,00	62,00
S2	17,00	0,13	322,00	14,00	44,00	64,00
S3	20,00	0,07	268,00	13,00	48,00	54,00
S4	21,00	0,05	257,00	18,00	43,00	53,00
S5	16,00	0,03	236,00	20,00	52,00	52,00
S6	20,00	0,09	245,00	23,00	45,00	43,00
S7	21,00	0,13	298,00	21,00	56,00	47,00
S8	19,00	0,07	255,00	18,00	45,00	42,00
S9	21,00	0,03	243,00	15,00	44,00	39,00
S10	24,00	0,03	235,00	19,00	43,00	36,00
S11	26,00	0,07	294,00	20,00	44,00	37,00
S12	22,00	0,08	263,00	21,00	45,00	35,00
S13	20,00	0,02	322,00	19,00	51,00	35,00
S14	18,00	0,11	232,00	18,00	48,00	39,00
S15	17,00	0,05	245,00	17,00	48,00	34,00
S16	22,00	0,04	211,00	17,00	51,00	36,00
S17	26,00	0,06	160,00	14,00	34,00	30,00
S18	30,00	0,07	210,00	19,00	55,00	41,00
S19	28,00	0,09	197,00	35,00	79,00	50,00
S20	22,00	0,05	247,00	38,00	64,00	53,00
Mean	21.90	0.06	251.20	19.85	49.50	4.10
SEM	0.88	0.01	9.05	1.39	2.08	2.17

Table S4. macronutrient, micronutrient, and HM concentrations in avocado leaves across orchard sites (S1 to S10).

Site	Tree	Macronutrient concentration (%)					Micronutrient concentration (mg/kg)					HM concentration (mg/kg)		
		N	P	K	Ca	Mg	B	Fe	Zn	Cu	Mn	Ni	Cd	Pb
S1	Tree 1	3.28	0.287	2.94	2.96	0.76	48.80	233.50	41.30	8.10	35.50	4.87	0.12	0.59
	Tree 2	2.80	0.256	2.57	2.45	0.87	45.50	240.00	44.80	8.50	33.20	4.39	0.12	0.41
	Tree 3	2.95	0.298	2.56	2.60	0.76	59.30	189.10	32.20	10.60	26.70	5.00	0.12	0.52
	Tree 4	3.22	0.305	2.79	2.63	0.74	42.60	218.90	35.20	11.10	34.50	4.22	0.10	0.40
S2	Tree 1	2.25	0.282	2.24	3.50	0.64	48.50	162.40	49.40	60.00	70.00	3.85	0.24	0.81
	Tree 2	2.65	0.269	2.23	3.50	0.54	51.80	136.80	44.30	10.10	70.00	4.81	0.16	0.75
	Tree 3	2.40	0.230	1.87	3.50	0.57	47.40	126.30	42.00	8.80	70.00	4.14	0.23	0.72
	Tree 4	2.57	0.256	1.83	3.50	0.67	51.50	122.70	44.50	6.50	70.00	4.19	0.15	0.77
S3	Tree 1	2.35	0.273	2.43	3.07	0.36	49.60	82.30	35.40	6.60	64.00	4.70	0.31	1.21
	Tree 2	2.15	0.288	2.09	3.19	0.57	57.00	100.70	38.40	10.90	46.70	4.52	0.28	1.45
	Tree 3	2.07	0.266	1.54	3.13	0.32	57.70	111.30	33.90	8.10	45.50	4.91	0.28	1.27
	Tree 4	2.42	0.270	2.10	2.79	0.59	52.60	95.30	36.80	7.20	48.30	4.92	0.33	1.46
S4	Tree 1	3.06	0.299	1.43	2.85	0.54	53.90	151.80	37.40	10.90	38.20	4.71	0.12	1.03
	Tree 2	2.53	0.272	1.54	2.71	0.56	51.40	124.10	41.60	11.90	34.80	5.00	0.24	0.92
	Tree 3	2.47	0.300	1.72	2.87	0.69	49.60	136.50	33.90	8.10	40.40	4.41	0.18	1.03
	Tree 4	2.76	0.272	1.83	2.73	0.70	51.80	165.90	46.90	11.10	29.30	3.83	0.22	0.80
S5	Tree 1	3.09	0.377	1.96	3.50	0.79	53.30	152.50	51.30	8.50	66.10	4.96	0.20	0.93
	Tree 2	2.62	0.314	1.30	3.25	0.55	50.30	149.20	43.90	11.40	63.60	5.00	0.15	0.91
	Tree 3	3.42	0.313	1.18	3.38	0.63	54.30	144.70	46.90	11.00	70.00	4.6	0.22	1.02
	Tree 4	2.86	0.335	1.91	3.09	0.79	60.60	154.20	39.90	9.00	70.00	4.85	0.22	0.88
S6	Tree 1	2.44	0.314	1.00	3.13	0.52	43.80	172.50	33.80	10.60	67.60	5.00	0.16	1.17
	Tree 2	2.63	0.295	1.98	3.38	0.77	50.30	145.30	41.60	12.80	70.00	3.88	0.19	0.93
	Tree 3	2.60	0.327	1.92	3.21	0.70	47.50	120.90	45.20	12.20	63.50	4.95	0.17	1.09
	Tree 4	2.75	0.298	1.67	3.49	0.59	49.90	146.30	47.40	10.30	69.70	4.40	0.11	1.19
S7	Tree 1	2.74	0.321	2.59	3.50	0.87	48.40	242.90	39.80	10.70	70.00	5.00	0.09	0.31
	Tree 2	2.33	0.328	2.43	3.50	0.73	49.90	185.40	40.90	9.10	70.00	5.00	0.06	0.36
	Tree 3	2.65	0.294	1.95	3.50	0.87	47.50	237.10	41.70	7.60	70.00	5.00	0.15	0.33
	Tree 4	2.87	0.322	2.15	3.50	0.98	49.90	235.40	42.10	8.80	70.00	5.00	0.14	0.32
S8	Tree 1	2.56	0.282	2.02	3.21	0.76	46.10	121.10	49.00	11.90	65.00	5.00	0.12	0.78
	Tree 2	2.46	0.334	1.71	3.04	0.75	39.40	122.80	42.70	9.30	61.90	5.00	0.20	0.54
	Tree 3	2.24	0.300	1.55	3.50	0.67	47.70	172.30	53.30	8.80	66.40	3.70	0.15	0.74
	Tree 4	2.43	0.281	2.14	2.99	0.67	50.60	156.60	50.10	6.80	52.50	5.00	0.12	0.89
S9	Tree 1	2.69	0.279	2.20	2.76	0.68	58.80	137.10	38.40	8.90	44.40	3.96	0.20	0.93
	Tree 2	2.03	0.253	1.23	2.70	0.55	57.50	125.30	41.70	5.00	29.00	4.42	0.13	1.17
	Tree 3	1.97	0.275	1.45	2.88	0.75	45.30	165.50	28.20	7.50	42.30	3.95	0.10	1.18
	Tree 4	2.22	0.274	1.53	2.64	0.58	59.80	148.90	30.00	8.10	37.00	4.33	0.18	0.77
S10	Tree 1	2.62	0.285	2.11	2.28	0.72	56.80	168.30	53.40	10.80	38.60	5.00	0.19	0.85
	Tree 2	2.53	0.264	2.25	2.59	0.74	46.80	124.70	63.10	11.40	37.10	5.00	0.13	0.86
	Tree 3	3.03	0.307	1.88	2.70	0.53	43.10	156.70	64.20	6.80	39.90	3.94	0.17	1.15
	Tree 4	2.55	0.297	1.94	2.80	0.60	48.80	161.50	51.70	11.10	39.60	3.77	0.15	1.08

Table S5. Macronutrient, micronutrient, and HM concentrations in avocado leaves across orchard sites (S11 to S20).

Site	Tree	Macronutrient concentration (%)					Micronutrient concentration (mg/kg)					HM concentration (mg/kg)		
		N	P	K	Ca	Mg	B	Fe	Zn	Cu	Mn	Ni	Cd	Pb
S11	Tree 1	2.35	0.31	1.99	3.35	0.63	41.00	177.80	54.20	10.70	57.30	4.71	0.22	0.82
	Tree 2	2.44	0.28	2.20	3.06	0.61	55.00	137.10	58.20	7.90	64.50	5.00	0.14	0.94
	Tree 3	2.80	0.29	2.30	3.12	0.69	49.20	178.60	55.30	10.80	59.80	4.15	0.17	0.71
	Tree 4	2.60	0.30	2.81	3.41	0.55	56.00	171.00	43.80	8.10	52.50	4.21	0.21	0.84
S12	Tree 1	2.56	0.30	2.30	2.96	0.68	53.90	146.10	48.50	11.20	68.20	4.81	0.18	0.63
	Tree 2	2.54	0.29	1.80	3.50	0.75	50.40	172.00	45.20	11.20	70.00	4.24	0.20	0.75
	Tree 3	2.41	0.31	2.28	3.22	0.66	56.80	166.20	52.20	8.90	64.00	3.61	0.23	0.68
	Tree 4	2.48	0.31	1.71	3.50	0.59	50.50	125.60	45.20	10.90	66.30	4.73	0.12	0.83
S13	Tree 1	2.78	0.28	1.77	3.06	0.72	55.50	133.70	49.30	9.40	52.00	4.56	0.23	0.81
	Tree 2	2.47	0.27	1.68	3.02	0.66	48.70	131.90	40.40	9.10	40.70	5.00	0.19	0.76
	Tree 3	2.83	0.31	1.99	2.86	0.57	50.30	128.50	41.50	10.10	53.40	4.78	0.10	0.81
	Tree 4	2.35	0.28	2.10	3.03	0.61	36.70	123.10	47.90	5.50	52.50	4.88	0.13	0.92
S14	Tree 1	2.69	0.32	2.11	2.46	0.73	44.40	126.90	43.50	6.40	14.00	4.64	0.19	0.65
	Tree 2	3.00	0.28	2.17	1.99	0.51	53.00	163.60	42.70	6.30	15.10	4.67	0.19	1.16
	Tree 3	2.77	0.26	1.36	2.33	0.71	58.80	135.80	49.50	9.30	10.00	4.49	0.21	0.75
	Tree 4	3.13	0.29	1.87	2.14	0.51	51.50	177.40	34.30	10.30	11.30	4.67	0.20	1.15
S15	Tree 1	2.46	0.26	1.85	2.00	0.67	41.50	171.50	42.60	8.80	14.70	5.00	0.18	0.77
	Tree 2	2.32	0.27	1.80	2.30	0.71	50.90	146.90	37.20	5.50	10.00	4.13	0.23	0.73
	Tree 3	2.38	0.33	1.94	2.23	0.63	53.50	125.10	40.90	13.40	10.00	5.00	0.24	0.53
	Tree 4	2.93	0.27	2.33	2.34	0.70	53.20	149.80	43.40	5.10	10.00	4.68	0.13	0.69
S16	Tree 1	2.83	0.25	1.91	2.28	0.64	55.40	130.50	41.80	8.20	10.00	5.00	0.20	0.73
	Tree 2	2.54	0.26	2.11	2.15	0.67	50.10	122.70	44.20	7.30	10.00	4.84	0.18	0.83
	Tree 3	2.84	0.27	2.17	1.98	0.62	47.80	152.20	40.50	10.40	10.00	5.00	0.14	0.75
	Tree 4	3.01	0.27	2.22	2.62	0.69	47.80	174.40	47.40	7.30	10.00	4.93	0.12	1.13
S17	Tree 1	2.12	0.25	1.67	2.11	0.37	45.00	81.20	26.20	6.70	10.00	2.85	0.35	1.30
	Tree 2	2.42	0.27	2.41	1.93	0.49	46.90	100.70	24.10	9.20	10.20	3.12	0.24	1.22
	Tree 3	2.21	0.28	1.74	1.76	0.57	59.00	99.70	25.30	7.40	10.00	3.74	0.28	1.16
	Tree 4	2.16	0.24	1.46	2.05	0.44	47.40	83.20	27.20	9.20	15.70	3.14	0.34	1.78
S18	Tree 1	2.51	0.33	2.08	2.57	0.53	55.50	104.60	44.80	11.40	38.20	5.00	0.28	1.51
	Tree 2	2.40	0.31	1.66	2.72	0.59	50.10	102.20	43.20	9.40	25.50	5.00	0.29	1.34
	Tree 3	2.31	0.29	1.98	2.96	0.47	51.40	90.80	43.20	8.40	33.30	5.00	0.30	1.50
	Tree 4	2.23	0.31	1.45	2.54	0.42	57.80	108.40	39.50	13.00	25.10	4.39	0.27	1.14
S19	Tree 1	2.21	0.38	1.86	2.03	0.73	54.40	151.80	58.10	21.10	10.00	5.00	0.18	0.55
	Tree 2	2.47	0.36	1.90	1.89	0.56	49.60	135.30	60.30	18.30	10.00	5.00	0.14	0.51
	Tree 3	2.46	0.38	1.76	2.43	0.59	59.00	176.50	51.50	17.70	10.00	5.00	0.13	0.83
	Tree 4	2.04	0.36	1.06	1.82	0.54	53.90	152.30	53.80	17.30	10.00	5.00	0.16	0.73
S20	Tree 1	2.62	0.39	2.60	1.96	0.99	46.60	250.00	80.00	22.80	21.60	5.00	0.13	0.33
	Tree 2	2.85	0.39	2.34	2.51	0.70	54.50	247.10	80.00	18.10	25.00	5.00	0.14	0.59
	Tree 3	3.01	0.39	2.34	2.27	0.75	52.10	209.10	80.00	19.20	29.10	5.00	0.13	0.56
	Tree 4	2.69	0.40	2.23	2.32	0.79	52.00	241.00	80.00	22.40	12.30	5.00	0.08	0.53

Table S6. Photosynthetic pigment profiles, spad index, chlorophyll a/b ratio, and leaf color status of avocado leaves across orchard sites (s1 to s10).

Site	Tree	SPAD Index	Chlorophyll a (µg/g FW)	Chlorophyll b (µg/g FW)	Leaf Color / Status	Chlorophyll a/b ratio
S1	Tree 1	49.40	67.30	34.90	Healthy green	1.93
	Tree 2	53.70	69.00	34.20	Healthy green	2.02
	Tree 3	47.80	65.90	39.10	Healthy green	1.69
	Tree 4	47.10	66.20	36.30	Healthy green	1.82
S2	Tree 1	42.30	42.00	27.20	Moderate (purple edges)	1.54
	Tree 2	44.10	42.70	22.40	Moderate (purple edges)	1.91
	Tree 3	44.70	42.70	28.50	Moderate (purple edges)	1.50
	Tree 4	39.90	43.70	28.70	Moderate (purple edges)	1.52
S3	Tree 1	24.50	25.10	13.60	Yellowing / chlorotic	1.85
	Tree 2	25.90	21.60	13.90	Yellowing / chlorotic	1.55
	Tree 3	29.70	22.60	16.60	Yellowing / chlorotic	1.36
	Tree 4	23.30	27.70	11.30	Yellowing / chlorotic	2.45
S4	Tree 1	44.70	46.80	22.40	Moderate (purple edges)	2.09
	Tree 2	35.70	42.50	25.20	Moderate (purple edges)	1.69
	Tree 3	38.40	52.40	24.30	Moderate (purple edges)	2.16
	Tree 4	37.50	49.30	27.10	Moderate (purple edges)	1.82
S5	Tree 1	36.70	42.50	20.40	Moderate (purple edges)	2.08
	Tree 2	42.40	50.00	24.70	Moderate (purple edges)	2.02
	Tree 3	43.40	52.10	25.90	Moderate (purple edges)	2.01
	Tree 4	43.70	43.10	21.10	Moderate (purple edges)	2.04
S6	Tree 1	37.70	40.90	25.30	Moderate (purple edges)	1.62
	Tree 2	44.40	40.60	21.20	Moderate (purple edges)	1.92
	Tree 3	39.50	54.00	23.20	Moderate (purple edges)	2.33
	Tree 4	40.10	40.60	21.50	Moderate (purple edges)	1.89
S7	Tree 1	54.90	69.70	30.00	Healthy green	2.32
	Tree 2	54.50	66.40	38.70	Healthy green	1.72
	Tree 3	49.50	65.20	34.90	Healthy green	1.87
	Tree 4	51.70	61.40	30.30	Healthy green	2.03
S8	Tree 1	38.10	50.60	22.00	Moderate (purple edges)	2.30
	Tree 2	41.70	54.50	20.90	Moderate (purple edges)	2.61
	Tree 3	41.70	46.70	28.70	Moderate (purple edges)	1.63
	Tree 4	36.80	50.40	28.40	Moderate (purple edges)	1.77
S9	Tree 1	44.40	50.20	25.00	Moderate (purple edges)	2.01
	Tree 2	41.20	53.00	25.70	Moderate (purple edges)	2.06
	Tree 3	35.30	54.00	26.90	Moderate (purple edges)	2.01
	Tree 4	41.80	43.20	26.60	Moderate (purple edges)	1.62
S10	Tree 1	38.90	49.80	21.10	Moderate (purple edges)	2.36
	Tree 2	41.60	55.00	20.50	Moderate (purple edges)	2.68
	Tree 3	44.80	46.10	28.70	Moderate (purple edges)	1.61
	Tree 4	42.80	48.50	27.40	Moderate (purple edges)	1.77

Table S7. Photosynthetic pigment profiles, spad index, chlorophyll a/b ratio, and leaf color status of avocado leaves across orchard sites (s11 to s20).

Site	Tree	SPAD Index	Chlorophyll a (µg/g FW)	Chlorophyll b (µg/g FW)	Leaf Color / Status	Chlorophyll a/b ratio
S11	Tree 1	43.80	46.10	23.30	Moderate (purple edges)	1.98
	Tree 2	41.70	52.10	27.60	Moderate (purple edges)	1.89
	Tree 3	43.00	46.50	28.20	Moderate (purple edges)	1.65
	Tree 4	36.20	48.20	20.10	Moderate (purple edges)	2.40
S12	Tree 1	38.20	45.50	24.00	Moderate (purple edges)	1.90
	Tree 2	42.00	45.80	24.50	Moderate (purple edges)	1.87
	Tree 3	37.40	45.60	22.30	Moderate (purple edges)	2.04
	Tree 4	35.70	49.10	26.70	Moderate (purple edges)	1.84
S13	Tree 1	41.20	47.00	23.80	Moderate (purple edges)	1.97
	Tree 2	43.60	47.80	24.80	Moderate (purple edges)	1.93
	Tree 3	35.30	45.10	23.80	Moderate (purple edges)	1.89
	Tree 4	39.00	48.70	25.30	Moderate (purple edges)	1.92
S14	Tree 1	41.10	51.50	28.10	Moderate (purple edges)	1.83
	Tree 2	42.20	54.30	20.20	Moderate (purple edges)	2.69
	Tree 3	37.00	40.10	26.50	Moderate (purple edges)	1.51
	Tree 4	44.00	43.70	29.30	Moderate (purple edges)	1.49
S15	Tree 1	35.60	54.00	23.50	Moderate (purple edges)	2.30
	Tree 2	36.00	47.30	22.60	Moderate (purple edges)	2.09
	Tree 3	37.80	44.60	28.00	Moderate (purple edges)	1.59
	Tree 4	40.40	44.70	26.10	Moderate (purple edges)	1.71
S16	Tree 1	42.20	44.10	24.10	Moderate (purple edges)	1.83
	Tree 2	36.20	42.70	26.80	Moderate (purple edges)	1.59
	Tree 3	36.80	47.90	27.10	Moderate (purple edges)	1.77
	Tree 4	36.10	48.50	22.60	Moderate (purple edges)	2.15
S17	Tree 1	29.60	24.80	18.10	Yellowing / chlorotic	1.37
	Tree 2	25.50	20.40	16.30	Yellowing / chlorotic	1.25
	Tree 3	29.50	26.00	18.20	Yellowing / chlorotic	1.43
	Tree 4	28.80	22.30	12.10	Yellowing / chlorotic	1.84
S18	Tree 1	26.10	24.10	18.40	Yellowing / chlorotic	1.31
	Tree 2	29.00	23.50	12.40	Yellowing / chlorotic	1.90
	Tree 3	27.80	22.70	18.20	Yellowing / chlorotic	1.25
	Tree 4	24.20	26.70	11.00	Yellowing / chlorotic	2.43
S19	Tree 1	41.20	46.80	25.90	Moderate (purple edges)	1.81
	Tree 2	36.70	51.10	28.60	Moderate (purple edges)	1.79
	Tree 3	37.20	41.40	20.20	Moderate (purple edges)	2.05
	Tree 4	41.40	49.10	25.50	Moderate (purple edges)	1.93
S20	Tree 1	47.30	63.90	35.90	Healthy green	1.78
	Tree 2	50.00	69.90	31.40	Healthy green	2.23
	Tree 3	52.00	64.00	34.30	Healthy green	1.87
	Tree 4	52.20	66.90	39.90	Healthy green	1.68

Table S8. BAFs of HMs in avocado leaves across of 20 orchard sites.

Site	Tree	BAF-Cd	BAF-Cu	BAF-Ni	BAF-Pb	BAF-Zn	BAF-Fe	BAF-Mn	Site	Tree	BAF-Cd	BAF-Cu	BAF-Ni	BAF-Pb	BAF-Zn	BAF-Fe	BAF-Mn
S1	1	1.34	0.45	0.10	0.01	0.55	18.80	10.82	S11	1	3.16	0.53	0.11	0.02	0.50	25.95	9.26
	2	1.43	0.47	0.09	0.01	0.60	19.32	10.12		2	2.03	0.39	0.11	0.03	0.54	20.01	10.43
	3	1.36	0.59	0.10	0.01	0.43	15.23	8.14		3	2.46	0.53	0.09	0.02	0.51	26.07	9.67
	4	1.13	0.62	0.08	0.01	0.47	17.62	10.51		4	3.04	0.40	0.10	0.02	0.41	24.96	8.49
S2	1	1.89	0.43	0.09	0.01	0.56	37.68	8.18	S12	1	2.30	0.53	0.11	0.02	0.57	26.47	9.71
	2	1.28	0.72	0.11	0.01	0.50	31.74	8.18		2	2.58	0.53	0.09	0.02	0.53	31.17	9.96
	3	1.80	0.63	0.09	0.01	0.47	29.30	8.18		3	2.95	0.42	0.08	0.02	0.61	30.11	9.11
	4	1.22	0.46	0.10	0.01	0.50	28.47	8.18		4	1.53	0.52	0.11	0.02	0.53	22.76	9.44
S3	1	4.43	0.51	0.10	0.02	0.50	22.65	12.38	S13	1	11.75	0.49	0.09	0.02	0.59	23.46	9.60
	2	4.09	0.84	0.09	0.03	0.54	27.72	9.03		2	9.65	0.47	0.10	0.02	0.49	23.14	7.51
	3	4.00	0.62	0.10	0.02	0.48	30.64	8.80		3	5.40	0.52	0.09	0.02	0.50	22.54	9.86
	4	4.77	0.55	0.10	0.03	0.52	26.23	9.34		4	6.90	0.28	0.10	0.03	0.58	21.60	9.69
S4	1	2.52	0.61	0.11	0.02	0.45	53.32	10.57	S14	1	1.76	0.36	0.10	0.02	0.53	38.99	13.38
	2	4.98	0.66	0.12	0.02	0.50	43.59	9.63		2	1.75	0.36	0.10	0.03	0.52	50.26	14.44
	3	3.72	0.45	0.10	0.02	0.41	47.95	11.18		3	2.01	0.53	0.09	0.02	0.60	41.72	9.56
	4	4.54	0.62	0.09	0.02	0.57	58.27	8.11		4	1.86	0.58	0.10	0.03	0.42	54.50	10.80
S5	1	6.93	0.43	0.10	0.02	0.54	53.72	9.82	S15	1	3.74	0.53	0.10	0.02	0.53	59.06	14.99
	2	5.13	0.57	0.10	0.02	0.46	52.55	9.45		2	4.70	0.33	0.09	0.02	0.46	50.59	10.20
	3	7.33	0.55	0.09	0.02	0.49	50.97	10.40		3	4.94	0.81	0.10	0.02	0.51	43.08	10.20
	4	7.47	0.45	0.09	0.02	0.42	54.31	10.40		4	2.70	0.31	0.10	0.02	0.54	51.58	10.20
S6	1	1.82	0.46	0.11	0.03	0.41	50.82	10.10	S16	1	5.20	0.49	0.10	0.02	0.53	44.24	12.84
	2	2.12	0.56	0.09	0.02	0.51	42.81	10.46		2	4.73	0.44	0.10	0.02	0.56	41.59	12.84
	3	1.98	0.53	0.11	0.03	0.55	35.62	9.49		3	3.73	0.63	0.10	0.02	0.51	51.59	12.84
	4	1.23	0.45	0.10	0.03	0.58	43.11	10.41		4	3.08	0.44	0.10	0.03	0.60	59.12	12.84
S7	1	0.72	0.51	0.09	0.01	0.49	35.00	7.89	S17	1	5.87	0.49	0.08	0.04	0.48	45.75	32.50
	2	0.51	0.43	0.09	0.01	0.50	26.71	7.89		2	4.12	0.67	0.09	0.04	0.44	56.73	33.15
	3	1.15	0.36	0.09	0.01	0.51	34.16	7.89		3	4.80	0.54	0.11	0.04	0.46	56.17	32.50
	4	1.13	0.42	0.09	0.01	0.52	33.92	7.89		4	5.75	0.67	0.09	0.06	0.50	46.87	51.02
S8	1	1.80	0.66	0.11	0.02	0.53	26.97	10.80	S18	1	4.10	0.61	0.09	0.04	0.53	25.98	11.81
	2	2.97	0.52	0.11	0.01	0.46	27.34	10.28		2	4.23	0.51	0.09	0.03	0.51	25.38	7.88
	3	2.21	0.49	0.08	0.02	0.57	38.37	11.03		3	4.30	0.45	0.09	0.04	0.51	22.55	10.30
	4	1.83	0.38	0.11	0.02	0.54	34.87	8.72		4	3.91	0.70	0.08	0.03	0.47	26.92	7.76
S9	1	6.87	0.58	0.09	0.02	0.57	39.80	11.83	S19	1	2.09	0.61	0.06	0.01	0.50	52.36	14.46
	2	4.37	0.33	0.10	0.03	0.62	36.37	7.73		2	1.61	0.53	0.06	0.01	0.51	46.67	14.46
	3	3.50	0.49	0.09	0.03	0.42	48.04	11.27		3	1.51	0.51	0.06	0.02	0.44	60.88	14.46
	4	6.10	0.53	0.10	0.02	0.45	43.22	9.86		4	1.81	0.50	0.06	0.01	0.46	52.54	14.46
S10	1	6.57	0.56	0.12	0.02	0.49	24.42	10.41	S20	1	2.68	0.59	0.08	0.01	0.42	24.15	11.72
	2	4.47	0.59	0.12	0.02	0.58	18.10	10.00		2	2.92	0.47	0.08	0.01	0.42	23.87	13.56
	3	5.77	0.35	0.09	0.03	0.59	22.74	10.76		3	2.78	0.50	0.08	0.01	0.42	20.20	15.79
	4	5.10	0.58	0.09	0.03	0.47	23.44	10.68		4	1.66	0.58	0.08	0.01	0.42	23.29	6.67

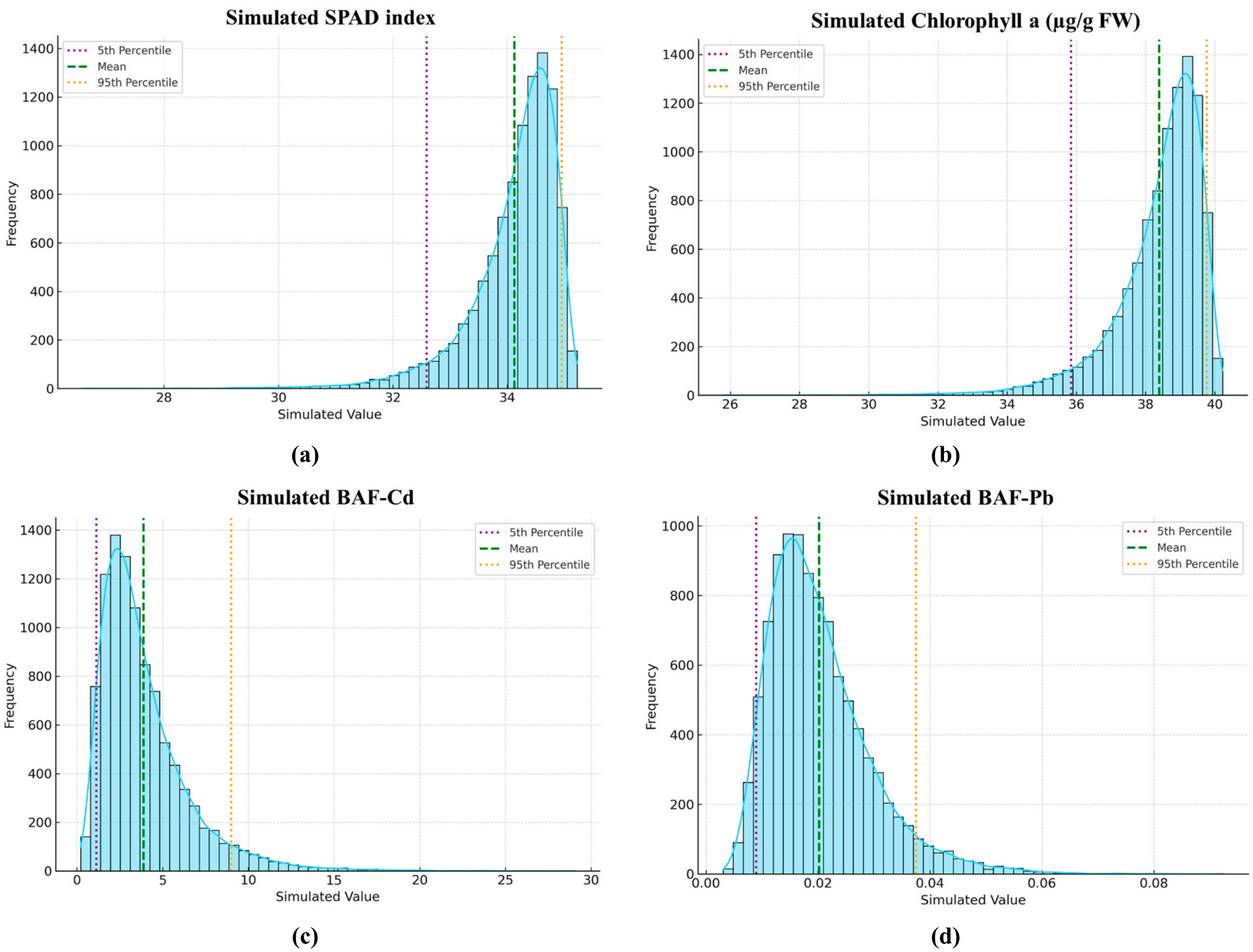

Figure S1. Probability distributions of simulated BAFs and photosynthetic indicators derived from MCS.
